# Redox non-innocent bis-silylene aluminium complexes with a carborane backbone†

**DOI:** 10.1039/d5sc01104c

**Published:** 2025-03-07

**Authors:** Artemis Saddington, Shenglai Yao, Christian Lorent, Matthias Driess

**Affiliations:** a Department of Chemistry: Metalorganics and Inorganic Materials, Technische Universität Berlin Strasse des 17. Juni 115, Sekr. C2 Berlin 10623 Germany matthias.driess@tu-berlin.de; b Department of Chemistry: Physical and Biophysical Chemistry, Technische Universität Berlin Strasse des 17. Juni 135, Sekr. PC14 Berlin 10623 Germany

## Abstract

The redox non-innocent bis-silylenyl *ortho*-carborane ligands [Si^II^(CC_cage_)Si^II^] (CC_cage_ = *o*-C_2_B_10_H_10_, Si^II^ = ArC(N^t^Bu)_2_Si; Ar = C_6_H_5_, *p*-^*t*^BuC_6_H_4_), with their particular chelating and electronic properties, have been employed for the synthesis of new donor-stabilized Si^II^ → Al^III^ complexes, potential precursors to low oxidation state aluminium complexes. Due to the redox non-innocence of the carborane backbone, [AlI_2_^+^] complexes with three ligand oxidation states were characterized: with neutral and radical anionic *closo*- as well as dianionic *nido*-C_2_B_10_ cores. Reduction at the aluminium center could also be enacted with potassium/naphthalene leading to {K[Si^II^(CC_cage_)Si^II^]Al(C_10_H_8_)} derivatives from [1 + 4] cycloaddition reaction. The mechanism of this dearomatization reaction is proposed to occur *via* the formation of transient low oxidation state aluminium intermediates (radicals and/or aluminylenes) that are trapped by naphthalene.

## Abstract 2

[AlI_2_^+^] complexes of a bis-silylenyl carborane have been synthesised with three interconvertible ligand oxidation states. Further reduction using K(C_10_H_8_) furnishes 1,4-naphthalene derivatives, indicative of low oxidation state Al intermediates.

## Graphical abstract

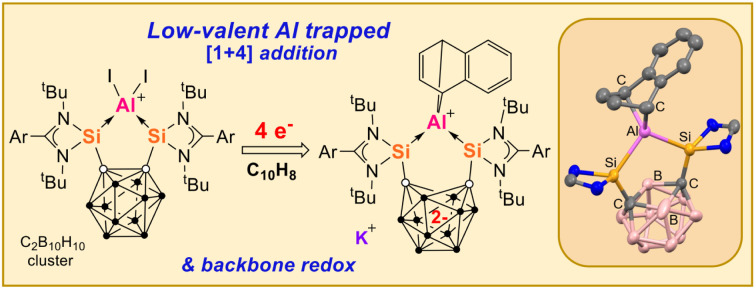

## Introduction

Strong σ-donors are an important tool in the synthesis of zero-valent main group element complexes, which have garnered significant interest in the last decade.^1–4^ Complexes of isolobal molecular ions supported by neutral ligands also rely on strong σ-donors for stabilization. Not only do such low valent compounds have interesting structures but they also possess unique potential applications. For example, they can act as ligands with unique bridging coordination modes, form otherwise difficult-to-realize molecules through oxidation, and have potential as novel single-source precursors for the synthesis of functional materials.^5–9^ Among the group 13 elements, the first zero oxidation state B_*n*_ (*n* = 1, 2, 4) compounds were isolated in recent years, mainly with carbene ligands.^10–14^ Of these, the rather π-accepting cAACs have also been applied to the synthesis of donor-stabilized aluminium compounds, such as an extraordinary mononuclear Al^I^ hydride reported by Braunschweig and coworkers, that also features significant diradical character at the carbene-C centers.^15–19^ Whilst no zero oxidation state aluminium compounds have been proposed yet, the first Al^I^ (aluminylene) cation I was synthesized in 2022 by Krossing and co-workers, with only neutral AlCp* substituents.^20^ Related Ga^I^ ion complexes with various donor ligands (*e.g.* PPh_3_, NHC) have been accessed from the useful salt complex [Ga(C_6_H_5_Me)_2_]^+^ [Al(OC(CF_3_)_3_)_4_]^−^ since 2010.^21,22^ In 2017, the first B^I^ (borylene) cation II (X = CO) was reported by Xie and Lin using a chelating bis-silylene ligand.^23^ We have demonstrated that bis-silylenes can act as effective σ-donor ligands, enabling access to monoatomic zero oxidation state group 14 and 15 complexes.^5,24–26^ Such compounds are capable of facile activation of small molecules such as H_2_, NH_3_, 9-BBN, CO and CO_2_.^27–30^ Silylenes are defined as divalent silicon species, isoelectronic with singlet carbenes.^31,32^ Amidinato-silylenes in particular can be fixed to many spacer molecules, establishing a family of highly adaptable σ-donating chelating ligands.^33–36^

**Fig. 1:**
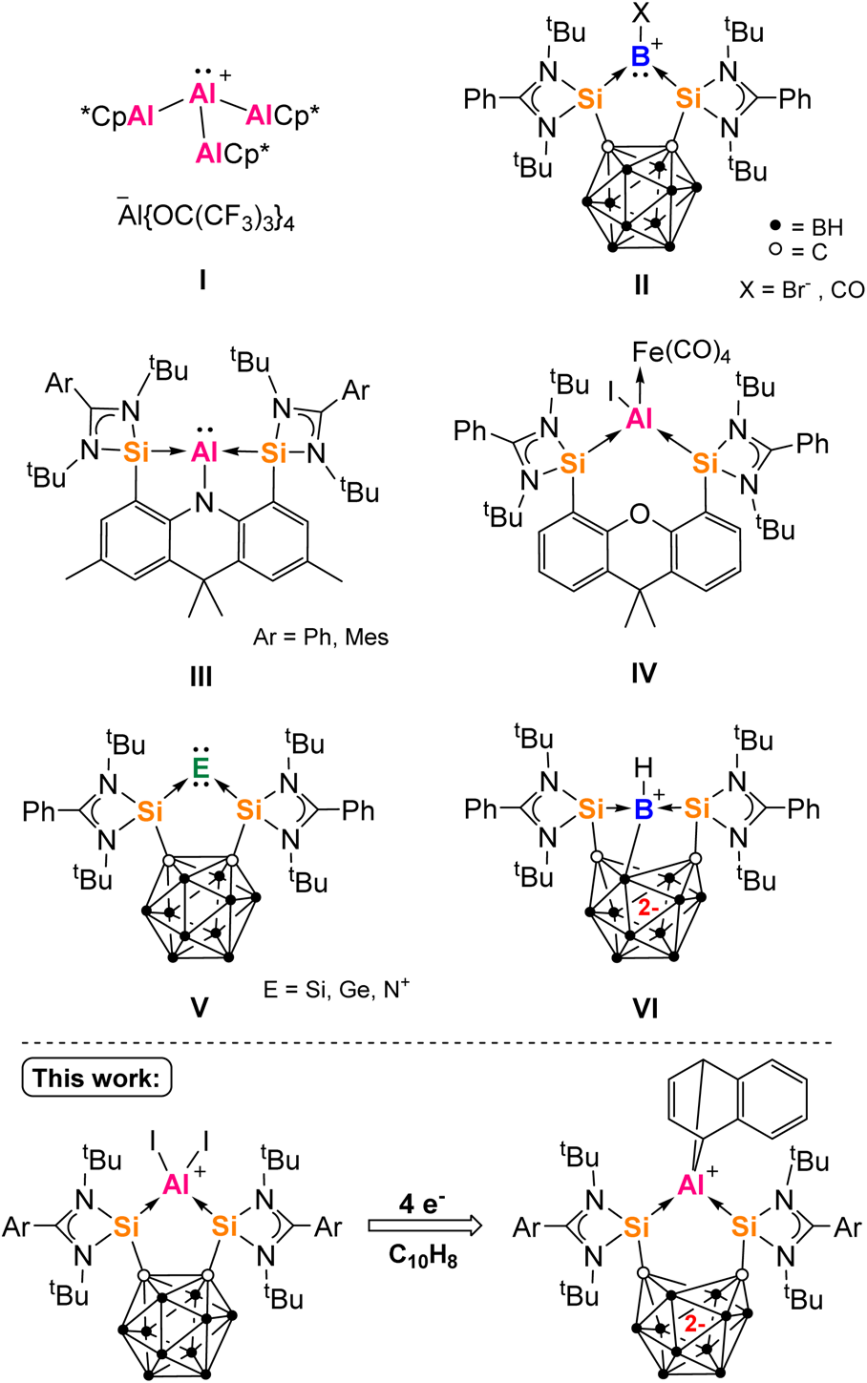
An Al(i) complex supported only by neutral ligands (I), examples of group 13–15 bis-silylene complexes (II–VI) and the new bis-silylene aluminium complexes of this work.

The area of low oxidation state silicon–aluminium chemistry is still in its infancy with the first examples of such compounds published in 2022–2023.^37–40^ Last year, we reported the transient pincer bis-silylene-supported aluminylene III (Ar = Ph), that dimerizes to give an Si_2_Al_2_ heterocycle (Fig. 1).^41^ Compound III could also be trapped as an iron(0) complex, from which we inferred its structure using density functional theory (DFT) calculations. Later, the isolable aluminylene III (Ar = Mes) was characterized by Mo and coworkers and agreed with our calculations.^42^ We also investigated the neutral bis-silylene [Si^II^(Xant)Si^II^] (Xant = 9,9-dimethylxanthene) for the synthesis of aluminylene complexes, but found it unsuitable for the synthesis of aluminylene compounds except as an Fe^0^ adduct IV.^41,43^

We turned our interest towards the bis-silylene [Si^II^(CC_cage_)Si^II^] 1a based on an *ortho*-icosahedral carborane spacer [CC_cage_ = *o*-C_2_B_10_H_10_, Si^II^ = LSi, L = PhC(N^*t*^Bu)_2_].^44,45^1a is a stronger σ-donor and π-acceptor ligand than [Si^II^(Xant)Si^II^], which has been demonstrated experimentally by their differing reactivity towards CO and P_4_, and relates to the inductive withdrawing effect of carborane substituents.^27,46–49^1a is also highly rigid, has a fixed smaller bite angle and generates more stable five-membered rings on complexation to a single atom. Furthermore, (C_2_B_10_)-functionalized compounds are capable of reversibly accepting one or two electrons to give radical anion or dianion complexes respectively.^50,51^ Accordingly, the two-electron reduction product {(K^+^(THF)_4_)_2_[Si^II^(CC_cage_)Si^II^]^2−^}_*n*_ has been isolated.^52^ We have used 1a to furnish some exceptional redox non-innocent complexes of Si^0^, Ge^0^ and N^I^VII, with other chelating carboranyl tetrylenes since being investigated.^48,52–58^ Complexes II and V (Fig. 1) are capable of redox-induced valence tautomerism or electron transfer (ET), rare for main group element complexes. This manifests as the unexpected flow of electrons in or out of the carborane cage away or towards the element center, often accompanied by E–E coupling.^59,60^ For example, upon two-electron reduction, II (X = Br^−^) becomes VI (Fig. 1).^61^ In this work, we investigated the suitability of 1 for the synthesis of primarily donor-stabilized aluminium compounds, that could potentially act as precursors to low oxidation state aluminium complexes. We were first able to isolate Al^III^ complexes of 1 with three different ligand oxidation states. Further reduction with K(C_10_H_8_) leads to 1,4-naphthalene derivatives of Al^III^, which points to the existence of largely unknown low-valent aluminium intermediates.

## Results and discussion

### Generation of bis-silylenyl carborane aluminium complexes

**Scheme 1:**
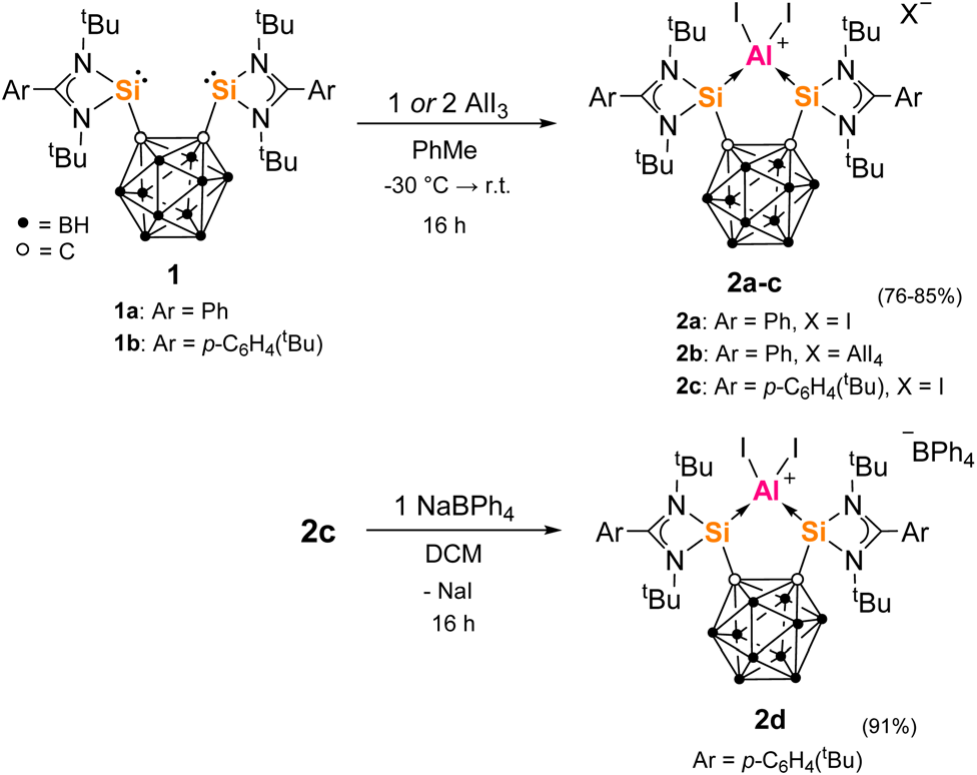
Synthesis of complexes 2a–c from bis-silylenes 1a-b and AlI_3_ and subsequent reaction of 2c with NaBPh_4_ to give 2d.

Many metallacarborane derivatives containing aluminium have been described since the 1970s.^62–65^ Despite chelating ligands with carborane scaffolds being well known, only recently did a report of an aluminium complex bearing a carborane backbone appear, specifically a *nido*-C_2_B_9_-based salen [NOON] ligand.^66,67^ We were able to prepare a series of *closo*-C_2_B_10_-based aluminium complexes straightforwardly using the bis-silylenes 1a and 1b (Scheme 1). Thus, adduct 2a was produced as colorless precipitate in 79% yield from the reaction of one molar equiv of 1a with one molar equiv AlI_3_ in toluene at low temperature. 2a exhibits very poor solubility in common aprotic solvents and decomposes instantly when dissolved in THF at room temperature. Its ^1^H and ^1^H/^13^C-coupled NMR spectra could therefore only be recorded in *o*-dichlorobenzene-D_4_. The marginally more soluble ion pair 2b was prepared similarly in 85% yield using two molar equivs of AlI_3_. A few colorless crystals of 2b were grown from Et_2_O solutions that were suitable for an scXRD analysis. Dissolution of 2b in THF led to an orange solution and almost clean reformation of the free bis-silylene 1a detected by ^1^H NMR.

**Fig. 2:**
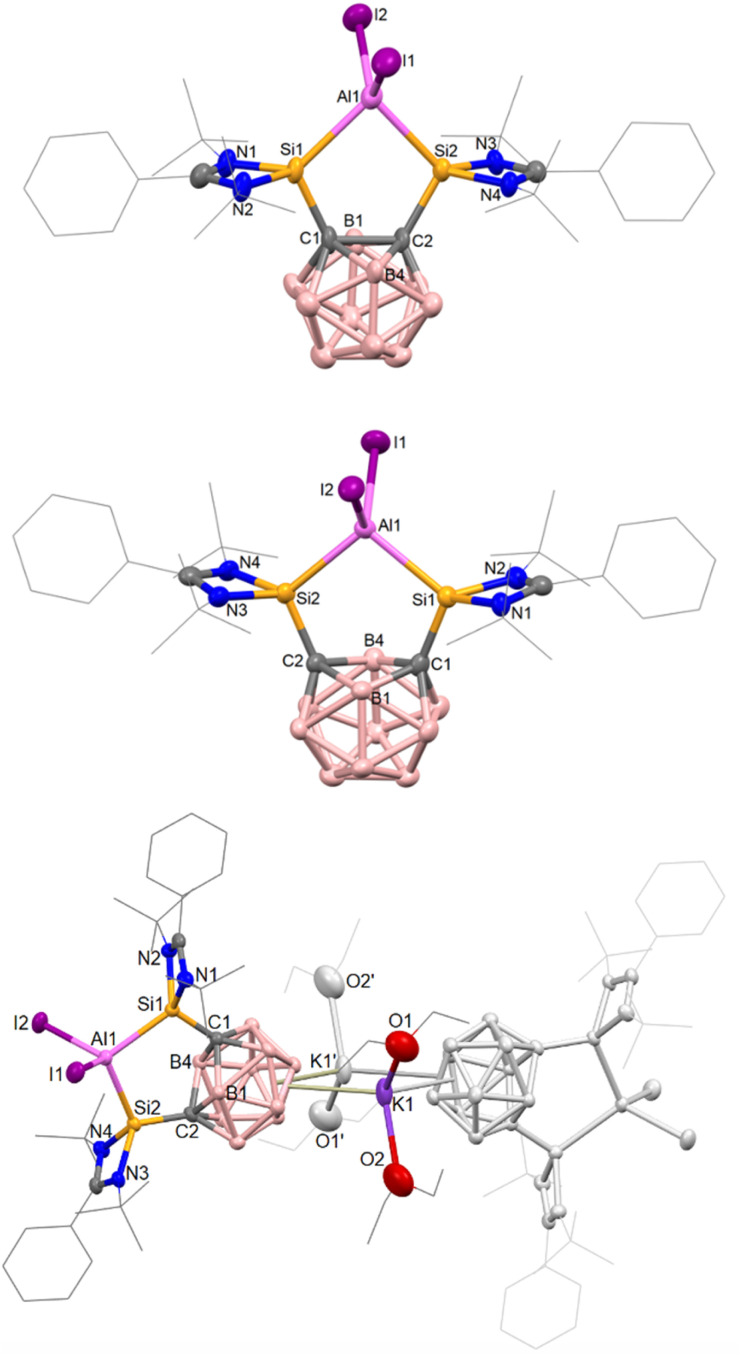
Molecular structures of the AlI_2_^+^-containing complexes of 2b (top, AlI_4_^−^ counterion omitted), 4a (middle) and [3(Et_2_O)_4_] (bottom) as determined by scXRD. Thermal ellipsoids have been set at 50% probability. Hydrogens and solvent molecules have been omitted and selected groups are shown in wireframe for clarity.

The cation of 2b contains a symmetrical C_2_Si_2_Al five-membered ring with an Si–Al–Si angle of 89.07(8)°, as determined by the rigid geometry of 1a, and with an average Si–Al bond length 2.493(3) Å (Fig. 2); the carborane C–C bond measures 1.729(8) Å. Compared to 2b, the isostructural complex [{(Si^II^(Xant)Si^II^)AlI_2_}I] has a much larger Si–Al–Si bond angle of 123.01(5)° but a similar average Si–Al bond length of 2.463(13) Å.^41^ Additionally, we later prepared the ^*t*^Bu-functionalized analogue 1b ([Si^II^(CC_cage_)Si^II^] with Si^II^ = L′Si, L′ = *p*-^*t*^BuC_6_H_4_C(N^*t*^Bu)_2_).^68^ The aluminium iodide adduct 2c was prepared in the same way as 2a and isolated as a pale-yellow precipitate in 76% yield (Scheme 1). 2c was characterized by multinuclear NMR spectroscopy in dichloromethane (DCM) before slowly decomposing overnight. Akin to 2a, 2c is also unstable in THF and still poorly soluble in acetonitrile. Where anion exchange reaction of 2a had failed using NaBPh_4_ and AgOTf when attempted in various solvents, we conveniently found that the iodide counterion of 2c could be exchanged simply in DCM. The reaction of one molar equiv 2c with one equiv NaBPh_4_ in DCM gives 2d as a pale-yellow solid in 91% yield. 2d is stable in DCM solution for weeks, whilst 2c is not. This indicates that the iodide counterion is not purely a spectator ion and potentially aids the relatively slower decomposition process to give free bis-silylene (compared to 2a in THF) which would go on to react with DCM (as known for silylenes).^69^

### Redox chemistry of the *o*-C_2_B_10_-cage in bis-silylenyl carborane M^III^ complexes (M = Al, Ga)

**Scheme 2:**
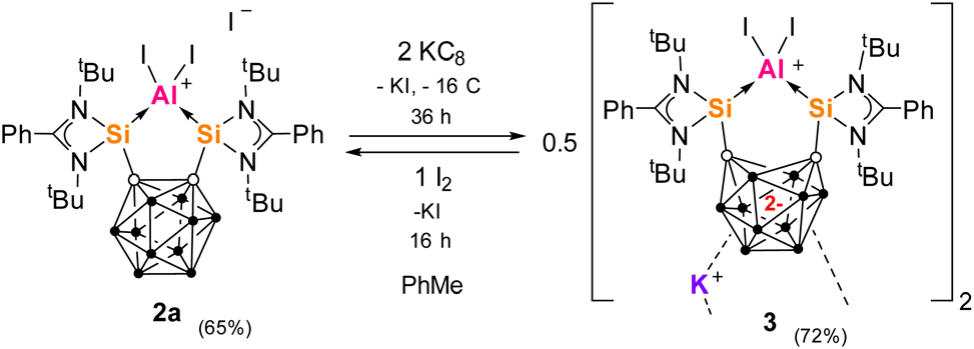
Two-electron reduction of 2a with KC_8_ to give dimer 3 and the reverse oxidation reaction with I_2_ to re-form 2a.

We first probed KC_8_ as a reducing agent, which has been successfully applied in the reduction steps to produce both II and III (Fig. 1). Reaction of one molar equiv 2a with a large excess KC_8_ (5 equivs) in toluene for 36 h leads to a bright red solution (Scheme 2). Dark pink needle-shaped crystals grew from the filtrate and were revealed by scXRD to be the zwitterionic dipotassium salt [3(C_6_H_5_Me)_2_], isolated in 72% yield. 3 was characterized by multinuclear NMR spectroscopy in THF but decomposed significantly within 2 hours. ^11^B{^1^H} NMR spectroscopy confirmed a change of environment for the B_10_ cage atoms with four signals at *δ*_B_ = −3.2, −7.2, −16.6 and −32.6 ppm [for 2c*δ*_B_ = 1.9, −3.5, −8.7 and −13.2 ppm (CD_2_Cl_2_)].

Crystals of [3(Et_2_O)_4_] suitable for scXRD analysis could be grown from Et_2_O solutions at low temperature (Fig. 2). In the ‘back-to-back’ dimeric structure of 2, the two K^+^ ions sit in-between the two carborane cages with the AlI_2_^+^ moieties facing outward. The reduction of the *o*-carborane moiety is apparent from the increased C⋯C distance. 3 features a *nido*-C_2_B_10_ cluster core with a C⋯C distance of 2.59 Å compared to 1.730(2) in 2b. Accompanying this change, the Si–Al–Si angle increases slightly from 89.08(7) to 101.99(6)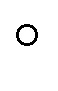. Other metal complexes of 1a, such as the Si^I^–Si^I^ and Ge^I^–Ge^I^ dimers (derived from V, Fig. 1) or dipotassium compound, have one-dimensional chain structures with K^+^ ions also bridging the dianionic *nido*-carborane moieties.^52,54^ The scXRD and ^11^B NMR data indicate that during reduction, two electrons are accepted by the C_2_B_10_ cage whilst the oxidation state of aluminium is unchanged.

3 can also be formed in an alternate synthesis, where 1a is reduced first with two equivs KC_8_ in THF (to give the dipotassium salt complex *in situ*) then reacted with one molar equiv AlI_3_ at low temperature. We propose that in the reaction of 2a with KC_8_, reduction begins with one electron being accepted by the unsaturated amidinato ligand which then moves to the carborane backbone to give a carborane radical anion intermediate. A similar mechanism for reduction of ligand 1a was demonstrated by DFT calculations investigating the reduction of silylone V.^52^ The initial formation of a transient aluminium radical intermediate is also possible. The formation of 3 contrasts with the formation of the related complexes of B^I^II and Si^0^, Ge^0^V, demonstrating how the *closo*-C_2_B_10_ cluster is reduced preferentially over Al^III^ but not over Si^II^, Ge^II^ or B^III^ under these reaction conditions.

We then investigated oxidation reactions of 3 that would release the two electrons from the *nido*-carborane cage. Test reactions with one or two equivs of AgOTf or FcPF_6_ (Fc = ferrocenium) in THF led to complex NMR spectra of unknown decomposition products. This can at least be in part explained by the instability of 2 in THF. 3 also decomposed in *o*-difluorobenzene (*o*-DFB), exhibited by a color change to dark brown (due to the formation of dark green precipitate). With polar solvents unsuitable, we used elemental iodine as an oxidizing agent. Thus, 0.5 molar equivs of dimer 3 were allowed to react with one molar equiv I_2_ in toluene, re-forming 2a in 65% isolated yield (Scheme 2). Attempts to determine the redox potentials of 2c in DCM by cyclic voltametric measurement were unsuccessful. Additionally, no K^+^ sequestration of 3 could be achieved with 18-crown-6 (18-c-6) or [2.2.2]-cryptand in THF. 3 also showed no reactivity towards NaBPh_4_ or CsBPh_4_. This lack of reactivity for 3 demonstrates that the K^+^ and I^−^ ions are strongly bound within the complex, to the carborane dianions and cationic aluminium centers respectively. In contrast, the structurally related N^I^ cation complex {K[Si^II^(CC_cage_)Si^II^]N} can form an ion-separated pair with 18-c-6 which is stable and undergoes one- and two-electron oxidation of the carborane cage with AgOTf.^53^

**Scheme 3:**
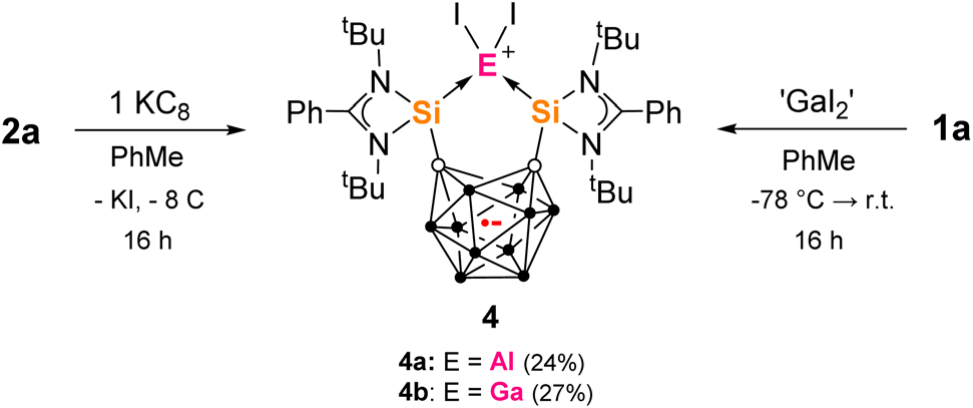
Formation of the bis-silylenyl-carborane radical complexes 4a and 4b from 2a and KC_8_ or 1a and ‘GaI_2_’ respectively.

**Fig. 3:**
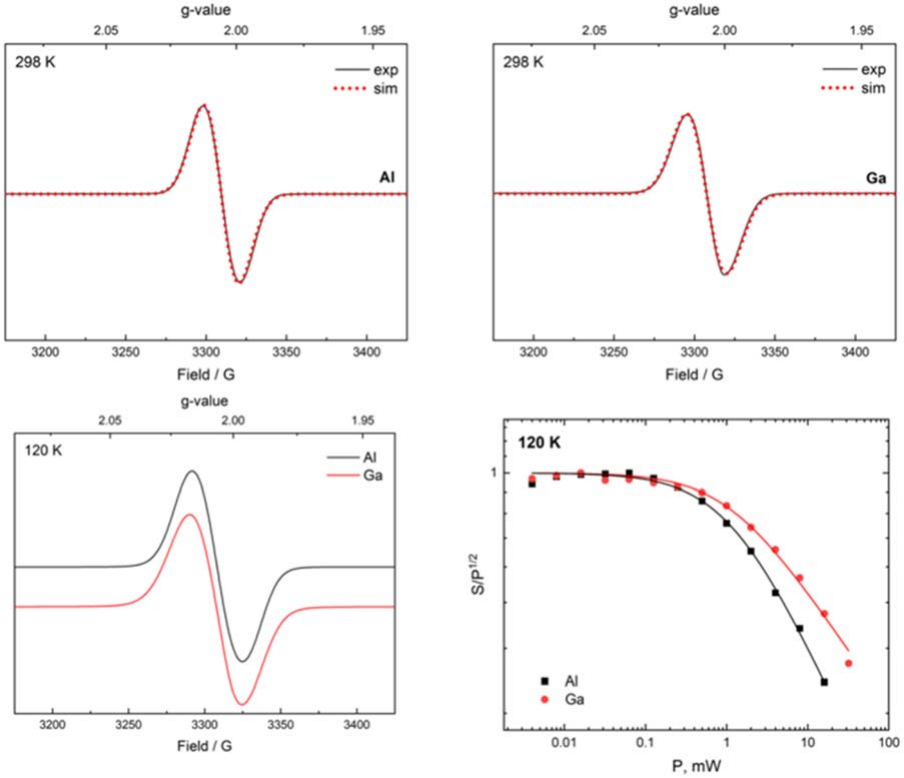
EPR data of the radical complexes 4a (top, left) and 4b (top, right) at room temperature. Cryogenic EPR spectra at 120 K (bottom, left) and the corresponding power saturation of 4a and 4b (bottom, right).

From the reaction of one molar equiv of 2a with a smaller excess of KC_8_ (2.5 equivs), the radical anion 4a, proposed as an intermediate in the formation of 3, was isolated in 24% yield (Scheme 3). The dark red crystals of 4a were suitable for scXRD analysis. The paramagnetic nature of 4a was evident from its NMR silence. EPR measurements at room temperature and below identified a strong isotropic radical signal at *g* = 2.007 and line width of 23.4 G, which suggests a delocalized carborane-based radical; no hyperfine coupling could be resolved (Fig. 3). In the solid-state structure of 4a, the C⋯C carborane distance is increased similarly to 3, at 2.43 Å (Fig. 2). The smaller carborane core opening agrees with the acceptance of one electron by the cluster to give a carboranyl radical anion. The geometry and bond distances around the Al center in 4a are generally similar to those in 3. Reduction of 4a with excess of KC_8_ (2.4 molar equivs) in Et_2_O also produces 3, additionally confirming 4a as a likely intermediate in the formation of 3 from 2a.

We were also able to isolate the gallium analogue 4b from the reaction of 1a with 1.4 molar equivs of ‘GaI_2_’ in toluene at low temperature (Scheme 3). ‘GaI_2_’ is a solid prepared from the reaction of elemental gallium and iodine in a 1 : 2 ratio.^70,71^4b could be isolated as a mixture of dark red and yellow crystals from red-orange toluene solutions in 27% yield. Radical 4b has an almost identical EPR signature to 4a with a strong isotropic radical signal at *g* = 2.008 and line width of 23.1 G, with no hyperfine coupling (Fig. 3). 4b and its water adduct [4b + (H_2_O)_5_] were additionally detected as molecular ions in mass spectrometry.

The scXRD analyses confirmed that both crystal types of 4b contain the same molecular structure. The yellow-colored crystals of 4b gave better metric data and the experimental bond lengths and angles were found to be almost identical to those of 4a (Fig. S36†). The different colored crystals of 4b result from different crystal packing and systems [yellow – orthorhombic, red – monoclinic]. The darker-colored crystals form predominantly from more concentrated solutions. In this two-fold reaction, the bis-silylenyl *o*-carborane accepts one electron and becomes bound to a GaI_2_^+^ fragment in the final product. The exact mechanism of how this might happen is unclear but overall, the ‘GaI_2_’ acts as a source of the GaI_2_˙ radical.

The EPR spectra of 4a and 4b recorded at 120 K verified that their isotropic radical signals are conserved at 120 K, exhibiting a small but significant difference in power saturation. The line shape, power and temperature dependency of 4a and 4b determined by EPR strongly suggest that the spin is delocalized in the C_2_B_10_ core. Whilst the spin of other paramagnetic aluminium complexes is typically ligand-based, such compounds tend to exhibit hyperfine coupling.^16,72–75^

With 2a, 3 and 4a in hand, we have established an interconvertible redox series of carborane-derived aluminium complexes, with three oxidation states characterized. This series based on 2a is unique for featuring an inorganic (carborane) electron storage unit (rather than aromatic/organic) that is not directly coupled or bonded to the Al center. The aluminum cation center is additionally only attached to the compound through donor bonds. There is a growing body of aluminium complexes with non-innocent ligands that can access several ligand oxidation states, all with charged N- or O-ligands, many of which are catalytically active.^76–79^ These complexes typically affect proton-coupled (PC)-ET or proton transfer (PT) reactions rather than ‘pure’ ET reactions, of which the few examples are rather diverse.^77,80–82^ We have demonstrated that carboranyl aluminium derivatives can act as reversible electron acceptors and that with further study have potential in catalytic ET reactions.

### Trapping of low valent aluminium with K(C_10_H_8_) 3

**Scheme 4:**
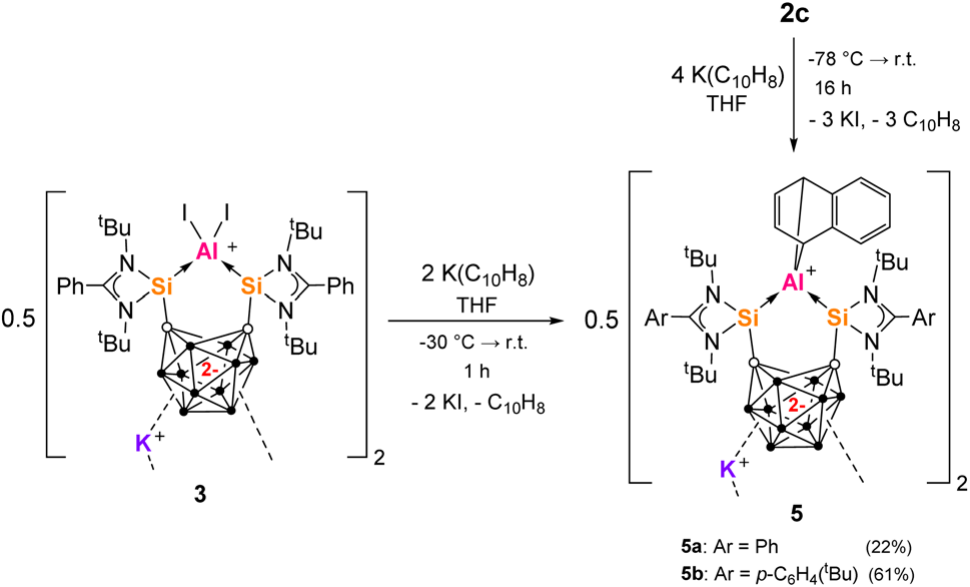
Formation of 1,4-naphthalene derivatives 5a and 5b from 3 or 2c and K(C_10_H_8_) respectively.

Thereafter, we wondered if further reduction of 3 would lead to a reduced aluminium center. With 3 in hand, we probed its reduction with excess KC_8_, also in the presence of 1,3-dienes, PCy_3_ or AlCl_3_. This only gave complicated mixtures resulting from significant decomposition. Therefore, we tested the reaction of 0.5 equivs of dimer 3 with two molar equivs K(C_10_H_8_) in THF in an NMR tube. The reagents reacted instantaneously on solvation forming a dark red solution and colourless precipitate. The ^1^H NMR spectrum showed the formation of one equiv of free C_10_H_8_ and one major species 5a (Scheme 4), with no decomposition observed after 3 weeks. In the scaled-up reaction, 5a was isolated as orange crystals from toluene in 22% yield. The ^1^H NMR spectrum of 5a strongly suggested that it was a 1,4-naphthalene derivative, with a characteristic set of multiplets (dd) at *δ*_H_ = 3.05, 6.38, 6.80 and 6.93 ppm. We attributed the low isolated yield of 5a to its very poor solubility in inert solvents (except THF). Therefore, we undertook a one-pot reaction with the potentially more soluble analogue 2c. One equiv of 2c was reacted with four molar equivs of K(C_10_H_8_) in THF at low temperature (Scheme 4). This furnished the expected product 5b, which was isolated as orange crystals from Et_2_O in 61% yield. 5a and 5b were characterized by multinuclear NMR spectroscopy in THF and benzene, respectively.

**Fig. 4:**
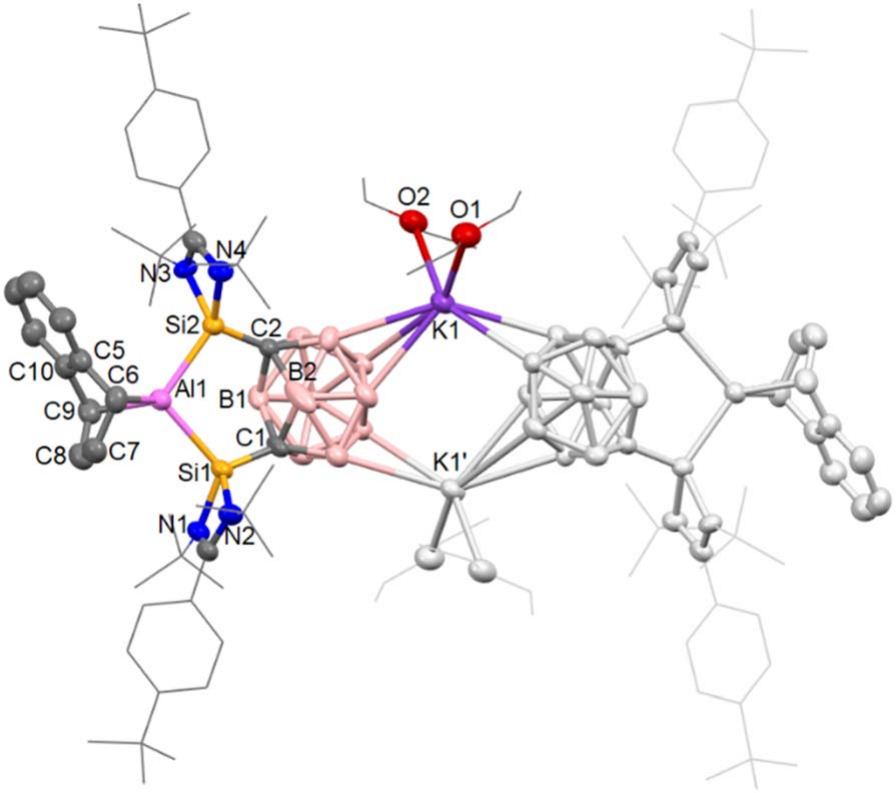
Molecular structure of [5b(Et_2_O)_4_]. Thermal ellipsoids have been set at 50% probability. Hydrogens and solvent molecules have been omitted and selected groups are shown in wireframe for clarity.

Akin to 3, 5a did not react with [2.2.2]-crypt, which might have aided the crystallization of single crystals of 5a. Eventually, after many crystallization attempts, suitable crystals of 5b from Et_2_O solution were obtained and measured by scXRD analysis (Fig. 4). The angles and distances measured for 5b are consistent with 3. The carborane C⋯C distance is maintained at approximately 2.60 Å as well as the Si–Al–Si bite angle at 100.95(5)°. The Al(C_10_H_8_) unit manifests as two Al–C bonds of lengths of 2.056(4) and 2.066(4) Å, with a C–Al–C angle of 77.49(17)°. The ^29^Si NMR spectrum of 5b revealed two resonances at *δ*_Si_ = 29.5 and 39.1 ppm. The non-equivalence of the ^29^Si nuclei can be explained by the orientation of the naphthalene group. 5a and 5b show consistent NMR and structural data with other Al(C_10_H_8_)-complexes resulting from Al^I^ activation of aromatics.^83–86^ This contrasts with the cooperative silylene-aluminylene reactivity of the pincer aluminylene III, which activates aromatic 2-methylquinoline through a 1,4-addition across the Al–Si bond instead.^42^

**Scheme 5:**
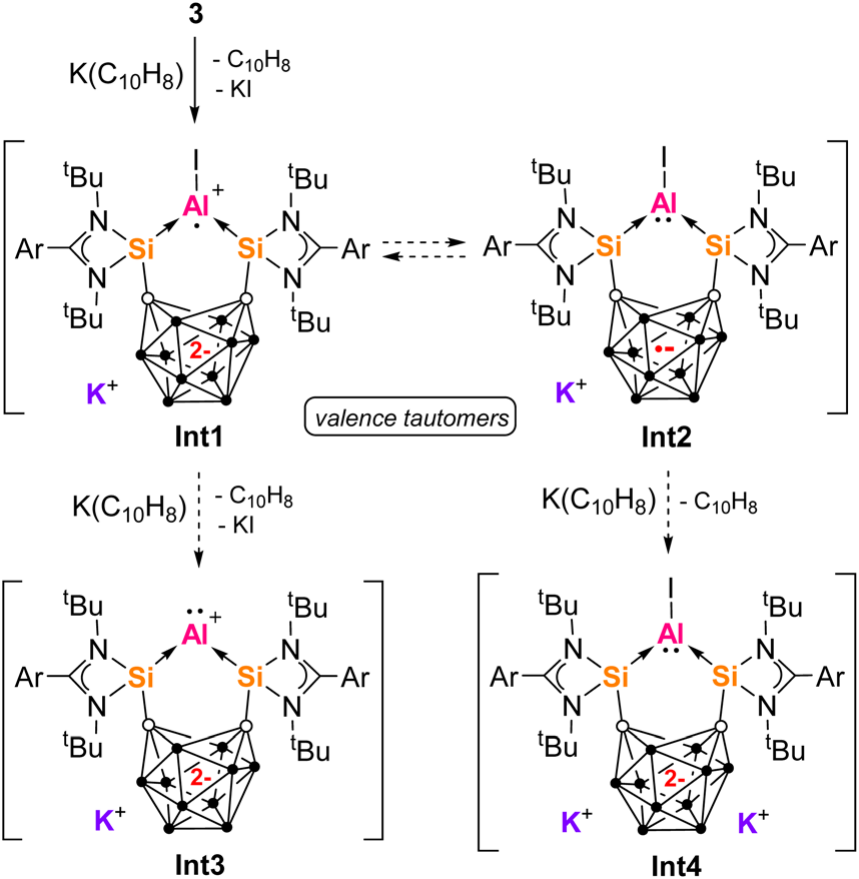
The structures of the fleeting aluminium intermediates Int1–Int4 proposed to form from one- or two-electron reduction.

The naphthalene-derived products 5a and 5b indicate a [1 + 4] cycloaddition reaction involving low oxidation state Al intermediates. Multiple reaction pathways are possible. Since 2c and 3 both generate the same product 5, we propose that 3 is also an intermediate in the reaction of 2c (Scheme 4). One-electron reduction of 3 with K(C_10_H_8_) would generate Int1 with an aluminium radical cation center first (Scheme 5). Due to presence of two electrons in the carborane backbone, one electron could be transferred to the Al center to give iodoaluminylene Int2 with an Al-based electron pair. Int1 and Int2 are valence tautomers or electromers. Int1 and Int2 would react with a second equivalent of K(C_10_H_8_) to generate aluminylene species Int3 and Int4 respectively. It is additionally possible than Int1 undergoes a radical cycloaddition with [C_10_H_8_]˙^–^ directly, while aluminylene species Int2–Int4 would undergo [1 + 4] cycloaddition with C_10_H_8_ generated in the reaction (as previously demonstrated by other aluminylene species).^84–86^ Such transient species as Int1–Int4 are unprecedented for aluminium, but have been proposed as boron intermediates *vide infra*. In a similar manner, transient boron(i) hydrides have also been captured as [1 + 2] cycloaddition products of naphthalene from reaction with Na(C_10_H_8_).^87,88^ Contrastingly, we did not observe C_10_H_8_ incorporation in our previous work with aluminium halide complexes of bidentate donor ligands [Si^II^(Xant)Si^II^] or a bis(NHC)-ligand (bis(*N*-dipp-imidazole-2-ylidene)methylene).^41,89^ Therefore this cycloaddition reactivity is unique to the Al complexes derived from ligand 1. We expect this difference is related to the stabilizing effects of the dianionic carborane backbone on the low-valent Al center, through countering the positive charge at Al or possibly even reducing the Al center through valence tautomerism.

Donor-stabilized haloaluminylenes have only been isolated as metal carbonyl complexes (*e.g.*IV, Fig. 1), while no donor-stabilized low valent aluminium cations have been reported.^41,89–92^ It is noted that a Al^I^ hydride [(cAAC)_2_AlH], Al^I^ cation [Al(AlCp*)_3_]^+^ (I, Fig. 1) and a silyl-substituted Al^III^ cation [R_3_Si-Al-SiR_3_]^+^ have been isolated and characterized.^15,20,93^ Radical cations, *e.g.* [AlH]˙^+^ and [AlF]˙^+^, have only been synthesized in matrix conditions.^94,95^ However, a neutral Al^III^ radical [(R_3_Si)_3_Al]˙ has been synthesized and mononuclear Al^II^ radicals [R_2_Al]˙ have also been recently detected *in situ* with EPR.^75,96^ Al^II^ radicals have also been proposed as intermediates in aluminium-directed reduction of H_2_, aromatics or alkynes.^83,97–99^ To our knowledge, electromerism (or valence tautomerism) resulting in the formation of an Al^I^ centre has not been reported.^59^ For the related boron complexes of ligand 1, species related to Int1–Int3 have been proposed as intermediates or isolated.^23^ One-electron reduction of bromoborylene II (Fig. 1) with Na(C_10_H_8_) gives an isolable B^II^ radical complex of ligand 1a (analogous to Int1), *via* the initial formation of its valence tautomer (analogous to Int2).^61^ Two-electron reduction of II yields VI (Fig. 1), with DFT calculations supporting the formation of a transient borylene cation (like Int3) that then reacts with a nearby cage B–H bond. Such a borylene or aluminylene cation like Int3 would be isolobal with the isolable zero-valent beryllium compound [(cAAC)_2_Be].^100^

Carbenes, specifically cAACs, have been used to tame or trap aluminium radical complexes *in situ*, where TEMPO is unsuitable.^16,19,75^ To find out more about the low-valent intermediates that form in the reaction to give 5a and 5b, we attempted reductions of 2c and 3 in the presence of ^Me^cAAC-5 with dropwise addition of THF solutions of crystalline [K(THF)(C_10_H_8_)] (of varying equivalents) at −78 °C (see ESI†).^17,101^ Formation of 5 was prevented but no cAAC-containing products were identified. To remove the option of C_10_H_8_ addition entirely, we carried out reduction reactions of 2 and 3 with K/KI (5% w/w) powder. Most promising was the reduction of the BPh_4_ salt 2d with one equiv ^Me^cAAC-5 and five equivs K/KI powder in Et_2_O (see ESI†).^102^ We observed color changes from colorless, *via* red, to dark blue within a few hours (Fig. S1 and S2†). ^1^H NMR aliquots of the reaction mixture in benzene showed no notable products, despite the maintaining the intense color for at least 2 weeks, and unfortunately, no viable single crystals for scXRD were isolated. Despite these experimental challenges, there is still more to uncover about the nature of such low valent Al species supported by 1. Further theoretical study could shine light on this and support the development of new ligand systems.

## Conclusions

Building on our previous work to access low oxidation state aluminium–silicon compounds, we employed the bis-silylenyl *o*-carborane ligands 1a and 1b to synthesize the respective bis-silylene Al^III^ complexes 2a–2d. Using KC_8_, the two-electron reduction of 2a could be achieved leading to the bis-silylenyl Al^III^*nido*-C_2_B_10_ dianion complex 3, whilst one-electron reduction affords the corresponding Al^III^ carborane radical anion complex 4a. Thus, we establish a redox series of aluminium complexes with three interconvertible oxidation states characterized. The analogous gallium radical complex 4b was also isolated from the reaction of 1a with ‘GaI_2_’. Using K(C_10_H_8_), the AlI_2_^+^ center of 3 or 2c was reduced furnishing the 1,4-naphthalene complexes 5a and 5b, respectively. Such products indicate the formation of transient donor-stabilized low-valent aluminium intermediates. These include the aluminium radical cation Int1 which could react further to form iodo- or cationic aluminylene intermediates Int2–Int4. Overall, it is evident that tunable chelating silylene ligands and redox non-innocent ligands have important roles to play in the continued development of donor-stabilized low oxidation state aluminium complexes towards zero valent aluminium.

## Data availability

All experimental data associated with this work are available in the ESI.†

## Author contributions

A. S. carried out the synthetic experiments, analyzed the experimental data and wrote the original manuscript. S. Y. carried out the scXRD refinement of the compounds and edited the manuscript. C. L. collected and analyzed the EPR data. M. D. supervised the work and edited the manuscript.

## Conflicts of interest

There are no conflicts to declare.

## Supplementary Material

SC-OLF-D5SC01104C-s001

SC-OLF-D5SC01104C-s002
