# Supplementary material for: Redox non-innocent bis-silylene aluminium complexes with a carborane backbone
Source: Chem Sci. 2025 Mar 7;16(15):6383–91. doi: 10.1039/d5sc01104c (PMC11907707; doi:10.1039/d5sc01104c)
Supplement: SC-016-D5SC01104C-s001 [file SC-016-D5SC01104C-s001.pdf]

Supplementary Information (SI) for Chemical Science.
This journal is © The Royal Society of Chemistry 2025

Supplementary Information

Redox Non-Innocent Bis-Silylene Aluminium Complexes with a Carborane Backbone

Artemis Saddington, Shenglai Yao, Christian Lorent and Matthias Driess*

Contents

1 General Considerations<br>• General methods and characterization<br>• Single crystal X-ray diffraction and structure determination<br>• EPR spectroscopy<br>• Preparation of starting materials and reagents	2-3
2 Synthesis and Characterization of compounds **1b-5**<br>• Attempts to trap fleeting low valent Al species	4-11
3 NMR Spectra	12-24
4 High Resolution Mass Spectrometry	25-28
5 Single Crystal X-Ray Diffraction Data	29-39
**References**	40

1 General Considerations

General methods and characterization

All experiments were carried out under dry oxygen-free nitrogen using standard Schlenk techniques or an MBraun inert atmosphere glove box under purified N_2 fitted with a gas purification and recirculation unit. Glassware was oven-dried at 120 °C for at least 16 h before use. Solvents were dried using standard methods and stored over 3 Å molecular sieves. THF, hexane and diethyl ether were obtained from an inert solvent purification system and then distilled from sodium/benzophenone. Toluene and dichloromethane were obtained from an inert solvent purification system and degassed with dry N_2. 1,2-difluorobenzene was dried over 3 Å molecular sieves. NMR spectra were recorded in benzene-D_6, THF-D_8, dichloromethane-D_2 or *o*-dichlorobenzene-D_4. THF-D_8 and benzene-D_6 were dried by distillation from sodium/benzophenone and stored over 3 Å molecular sieves. Dichloromethane-D_2 and *o*-dichlorobenzene-D_4 were dried and stored over 3 Å molecular sieves.

The solution ^{1}H, ^{13}C, ^{11}B and ^{29}Si NMR spectra were recorded on Bruker Spectrometers AV 400 or 500 with residual solvent signals as internal reference (^{1}H and $^{13}C\{^{1}H\}$ NMR) or external standards (^{11}B and $^{11}B\{^{1}H\}$ NMR: $BF_3.OEt_2$, 0.0 ppm; $^{29}Si\{^{1}H\}$ NMR: $SiMe_4$, 0.0 ppm). ^{27}Al NMR signals were too broad to be observed. Generally, ^{29}Si NMR signals were also not observed, even with H,Si-HMQC experiments. High-resolution MS spectra were measured on a Thermo Scientific LTQ orbitrap XL. Elemental analyses were performed on a Flash EA 1112 CHNS Analyzer by the analytical service at the Institute of Chemistry, Technical University of Berlin, Germany. Satisfactory elemental analysis could not be achieved for compounds **3**, **4a**, **5a** and **5b**.

Single crystal X-ray diffraction and structure determination

The crystals were mounted on a glass capillary in per-fluorinated oil and measured in a cold N_2 flow. The data of **2b**, **3**, **4a**, **4b** and **5b** were collected on an Oxford Diffraction Supernova, Single source at offset, Atlas at 150 K (Cu- Kα-radiation, λ = 1.5418 Å). The structures were solved with the SHELXT[1] and refined with Olex2[2,3] software package. The positions of the H atoms were calculated and considered isotropically according to a riding model. In the molecular structure of compound **2b**, two of the iodine atoms are disordered over two orientations with an occupancy ratio of 0.55:0.45. One THF molecule can be found in the asymmetric unit, but it is severely disordered and has been removed with the solvent-mask procedure in Olex2. In the single crystal containing compound **4a**, one toluene molecule can be found in the asymmetric unit, but it is severely disordered and has been removed with the solvent-mask procedure in Olex2. In the single crystal containing compound **4b**, two toluene molecules can be found in the asymmetric unit, but they are severely disordered and have been removed using the squeeze routine of platon program. In the molecular structure of compound **5b**, two THF molecules can be found in the asymmetric unit, but they are severely disordered and have been removed with the solvent-mask procedure in Olex2. CCDC deposit numbers for the reported compounds are as follows: 2418300 (compound **2b**), 2418301 (compound **3**), 2418298 (compound **4a**), 2418297 (compound **4b**) and 2418302 (compound **5b**). The supplementary crystallographic data for

this paper can be obtained free of charge from The Cambridge Crystallographic Data Centre via www.ccdc.cam.ac.uk/structures/.

EPR Spectroscopy

The EPR spectra were accumulated on a Bruker EMXplus spectrometer equipped with an ER 4122 SHQE resonator. Experimental conditions: 1 mW microwave power, microwave frequency: 9.3 GHz, 2 G modulation amplitude, 100 kHz modulation frequency. The simulation of the spectrum was performed using the MATLAB toolbox EasySpin (version 5.2.25).[4] The power saturation data was fitted to an empirical equation as described by Hirsh et al.[5]

Preparation of starting materials

Bis-silylene **1a** was prepared from 1,2-$C_2B_{10}H_{12}$ and LSiCl according to literature procedures.[6,7] The modified chlorosilylene (*p*-(^{t}Bu)C_6H_4)C(N^{t}Bu)$_2$SiCl was synthesized according to literature procedure from *p*-(^{t}Bu)C_6H_4Br.[8] Potassium graphite (KC_8) was prepared by reacting potassium with previously dried graphite in a 1:8 molar ratio at 160 °C for 2 h. Potassium naphthalenide was prepared by reacting potassium with one molar equiv naphthalene crystals in THF and stirred 12 h. The solvent was removed *in vacuo* giving K($C_{10}H_8$) as a black powder to be stored in the glovebox. Crystalline [K($C_{10}H_8$)(THF)] was prepared according to literature procedure.[9] K/KI (5% w/w) was prepared according to literature procedure.[10] The ^{Me}cAAC-5 carbene was prepared according to literature procedure.[11]

Aluminium iodide (AlI_3) was prepared by reacting iodine (I_2) crystals (1.5 equivs) with aluminium metal ribbon (1.1 equivs) in toluene at 60 °C for at least 3 h (until it turned colorless).[12] The hot solution was decanted and microcrystalline AlI_3 was crashed out with cooling then separated and dried *in vacuo*. Yield: 87% (4.6 g).

Gallium diiodide 'GaI_2' was prepared from the reaction of Ga metal (1 equiv) with I_2 (1 equiv) in toluene at 60 °C for at least 3 h (until the solvent turned colorless).[13,14] The toluene was removed *in vacuo* and the 'GaI_2' solid isolated as a pale-green powder. Yield: 90% (4.0g).

A2 Synthesis and Characterization of compounds 1b – 5

Compound **1b**

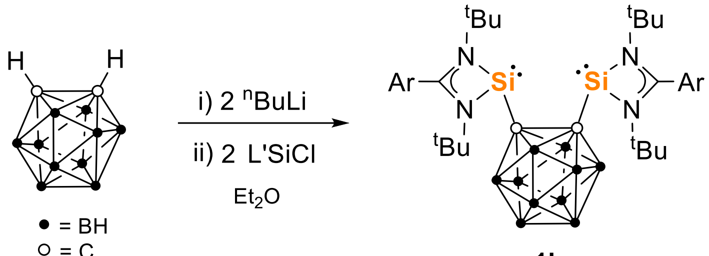

1b (85%)

Ar = *p*-C_6H_4(^{t}Bu)

Compound **1b** was prepared in the same manner as the reported compound **1a** in 85% yield, using silylene (*p*-(^{t}Bu)C_6H_4)C(N^{t}Bu)$_2$SiCl (L'SiCl) instead of PhC(NtBu)$_2$SiCl (LSiCl).

^{1}H NMR (500 MHz, C_6D_6, 298 K, ppm) *δ* = 7.37 – 7.33 (m, 2H, Ar*H*), 7.09 – 7.02 (m, 6H, Ar*H*), 3.89 – 2.60 (br., 10H, B*H*), 1.30 (s, 36H, NC(C*H*$_3$)$_3$), 1.12 (s, 18H, Ar-C(C*H*$_3$)$_3$). **^{13}C{^{1}H} NMR** (126 MHz, C_6D_6, 298 K, ppm) *δ* = 168.7 (N*C*N), 153.3, 131.8, 130.7, 128.6, 128.1, 125.2, 123.9, (Ar*C*), 85.6 (*C*Si), 54.1 (N*C*(CH$_3$)$_3$), 34.7 (Ar-*C*(CH$_3$)$_3$), 32.2 (NC(*C*H$_3$)$_3$), 31.2 (Ar-C(*C*H$_3$)$_3$). **^{11}B NMR** (161 MHz, C_6D_6, 298 K, ppm) *δ* = 0.8 (d, J_{BH} = 88.9 Hz), -5.0 (d, J_{BH} = 127.5 Hz), -9.4 (br.). **^{29}Si{^{1}H} NMR** (99 MHz, C_6D_6, 298 K, ppm) *δ* = 19.1. **HRMS** (APCI) *m/z* calcd for $C_{40}H_{72}B_{10}N_4Si_2$+3(OH): 823.6367 [*M*+3(OH)]; found: 823.6375.

Compounds **2a**, **2b**, **2c**

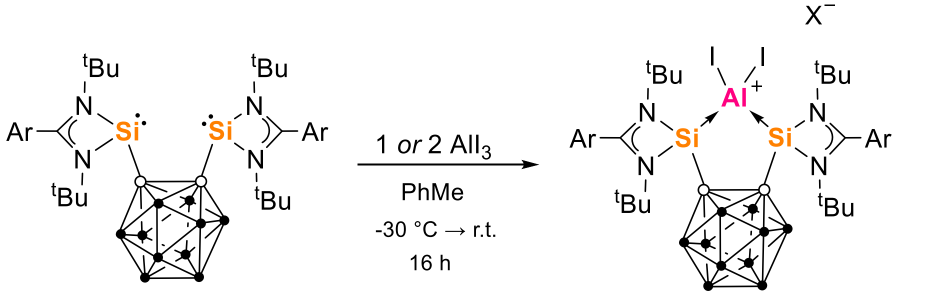

1
1a: Ar = Ph
1b: Ar = *p*-C_6H_4(^{t}Bu)

2a,b,c (76-85%)
2a: Ar = Ph, X = I
2b: Ar = Ph, X = AlI_4
2c: Ar = *p*-C_6H_4(^{t}Bu), X = I

General procedure:

o-carboranyl-bis(silylene) **1a** or **1b** was mixed with AlI_3 in a Schlenk flask. The mixture was cooled to -30 °C and toluene was added with rapid stirring. The reaction flask was allowed to slowly warm to room

temperature and stirred overnight (ca. 16 h). On warming, the golden color of the bis(silylene) disappeared and precipitate formed, giving a pale-yellow suspension. The precipitate was separated by cannula filtration, washed with Et_2O and then dried under vacuum for 2 h at 50 °C.

2a: Ligand **1a** (1.10 g, 1.66 mmol) was reacted with AlI_3 (0.685 g, 1.68 mmol, 1.0 equiv) in toluene (30 mL) to give **2a** as a colorless powder (1.41 g, 79%).

^{1}H NMR (400 MHz, *o*-DCB-d$_4$, 298 K) δ =7.54 (d, $^3J_{HH}$ = 7.5 Hz, 2 H, Ph*H*), 7.22 – 7.38 (m, 8 H, Ph*H*), 2.30 – 3.47 (br., 10H, B*H*), 1.36 (s, 36 H, C(C*H*$_3$)$_3$). **^{1}H,^{13}C-HSQC NMR** (400 MHz, *o*-DCB-d$_4$, 298 K) δ_C = 131.5, 128.2, 128.2, 128.1, 128.0 (*C*$_6$H$_5$), 31.3 (C(*C*H$_3$)$_3$). **^{1}H,^{13}C-HMBC NMR** (400 MHz, *o*-DCB-d$_4$, 298 K) δ_C = 129.1 (*i*-*C*$_6$H$_5$), 56.2 (*C*(CH$_3$)$_3$). Elemental analysis calculated for $C_{32}H_{56}B_{10}N_4Si_2AlI_3$: C 35.96, H 5.28, N 5.24; found: C 34.94, H 6.13, N 4.81.

2b: Ligand **1a** (0.500 g, 0.76 mmol) was reacted with AlI_3 (0.616 g, 1.51 mmol, 2 equiv), giving **2b** as a colorless powder (0.960 g, 85%). Crystals suitable for scXRD analysis were grown from a saturated Et_2O solution at -22 °C in 2 d.

Elemental analysis calculated for $C_{32}H_{56}B_{10}N_4Si_2Al_2I_6$: C 26.03, H 3.82, N 3.79; found: C 25.91, H 4.08, N 3.56.

2c: Ligand **1b** (1.52 g, 1.97 mmol) was reacted with AlI_3 (0.80 g, 1.96 mmol, 1 equiv), producing **2c** as a pale-yellow solid (1.77 g, 76%).

^{1}H NMR (500 MHz, CD_2Cl_2, 298 K, ppm) δ = 7.60 (d, $^3J_{HH}$ = 7.5 Hz, 2H, Ar*H*), 7.53 (d, $^3J_{HH}$ = 8.4 Hz, 2 H, Ar*H*), 7.32 (d, $^3J_{HH}$ = 7.3 Hz, 2H, Ar*H*), 2.02 – 3.38 (br., 10H, B*H*), 1.38 (s, 36H, NC(C*H*$_3$)$_3$), 1.36 (s, 18H, Ar-C(C*H*$_3$)$_3$). **^{13}C{^{1}H} NMR** (126 MHz, CD_2Cl_2, 298 K, ppm) δ = 181.3 (N*C*N), 156.1, 129.6, 128.8, 127.4, 125.6, 125.6 (Ar*C*), 76.7 (*C*Si), 57.2 (N*C*(CH$_3$)$_3$), 35.6 (Ar-*C*(CH$_3$)$_3$), 32.3 (NC(*C*H$_3$)$_3$). 31.5 (Ar-C(*C*H$_3$)$_3$). **^{11}B{^{1}H} NMR** (126 MHz, CD_2Cl_2, 298 K, ppm) δ = 1.9, -3.5, -8.7, -13.2. Elemental analysis calculated for $C_{40}H_{72}B_{10}N_4Si_2AlI_3$: C 40.68, H 6.15, N 4.74; found: C 40.17, H 5.80, N 4.41.

Compound **2d**

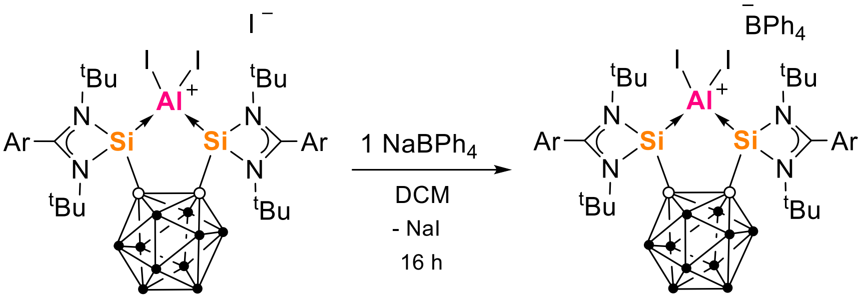

2c

Ar = *p*-$C_6H_4(^tBu)$

2d (91%)

Compound **2c** (400mg, 0.34 mmol) was loaded in a Schlenk flask with $NaBPh_4$ (122 mg, 0.36 mmol, 1.05 equiv) and then dissolved in dichloromethane (20 mL). The mixture was stirred overnight (ca. 16 h) to ensure full dissolution of $NaBPh_4$, giving a colorless cloudy mixture. The solvent was removed *in vacuo*, washed with hexane and the resulting pale-yellow solid was dried for 1 h at 55 °C, giving **2d** (423 mg, 91%).

^{1}H NMR (500 MHz, CD_2Cl_2, 298 K) δ = 7.64 (td, $^3J_{HH}$ = 8.1 Hz, $^4J_{HH}$ = 1.4 Hz, 4H, Ar*H*), 7.47 (dd, $^3J_{HH}$ = 8.1 Hz, $^4J_{HH}$ = 1.9 Hz, 2H, Ar*H*), 7.35 (dd, $^3J_{HH}$ = 8.1 Hz, $^4J_{HH}$ = 1.9 Hz, 2H, Ar*H*), 7.33 – 7.28 (m, 8H, B-Ph*H*), 7.02 (t, $^3J_{HH}$ = 7.4 Hz, 8H, B-Ph*H*), 6.87 (t, $^3J_{HH}$ = 7.1 Hz, 4H, B-Ph*H*), 3.21 – 2.11 (br., 10H, B*H*), 1.39 (s, 18H, Ar-C(C*H*$_3$)$_3$), 1.35 (s, 36H, NC(C*H*$_3$)$_3$). **^{13}C{^{1}H} NMR** (126 MHz, CD_2Cl_2, 298 K) δ = 185.9 (N*C*N), 165.2, 164.8, 164.4, 164.0, 157.6, 136.4, 136.4, 130.0, 127.4, 126.4, 126.2, 126.2, 126.2, 126.2, 125.4, 122.3 (Ar*C*), 58.3 (N*C*(CH$_3$)$_3$), 35.8 (Ar-*C*(CH$_3$)$_3$, 32.3 (NC(*C*H$_3$)$_3$), 31.4 (Ar-C(*C*H$_3$)$_3$). **^{11}B NMR** (161 MHz, CD_2Cl_2, 298 K) δ = 3.5 (br., *B*H), -1.1 (br., *B*H), -6.6 (s, *B*Ph$_4$), -9.0 (br., *B*H), -13.1 (br., *B*H).

Compound **3**

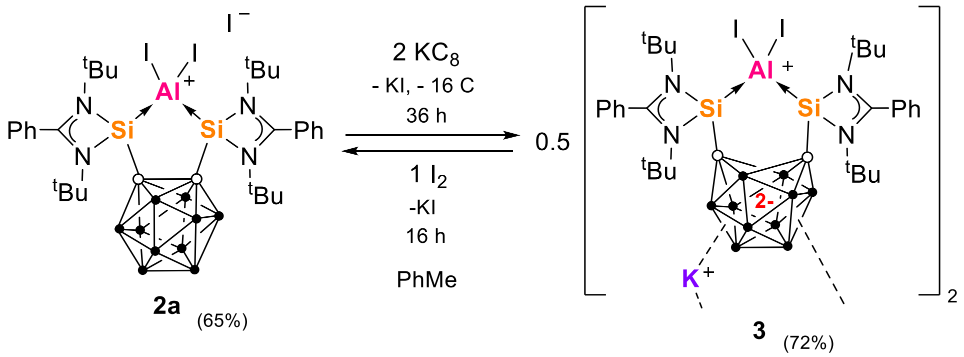

2a (65%) **3** (72%)

<u>Reduction of **2a**</u>

Compound **2a** (0.700 g, 0.65 mmol) was mixed with excess KC_8 (0.44 g, 3.25 mmol, 5 equiv) in a Schlenk flask and then suspended in toluene (50 mL). The suspension was stirred for 36 h to give a dark red-brown suspension. The suspension was allowed to settle then cannula filtered to give a bright red solution. The solution was concentrated by 50% and stored at -22 °C. After storage in the freezer for 2 d, red needle-like crystals formed. The crystals were confirmed by scXRD analysis to be [**3**(C_6H_5Me)$_2$]. The crystals were separated from the supernatant by decanting and dried for 2 h at 45 °C to give **4** as a pink powder (458 mg, 72%). Crystals of [**3**(Et_2O)$_4$] suitable for scXRD analysis were grown from a concentrated Et_2O solution stored at -22 °C overnight.

^{1}H NMR (500 MHz, THF-d$_8$, 298 K) δ = 7.59 (d, $^3J_{HH}$ = 7.5 Hz, 2H, Ph*H*), 7.56 – 7.45 (m, 8H, Ph*H*), 1.31 (s, 36 H, C(C*H*$_3$)$_3$). **^{13}C{^{1}H} NMR** (125 MHz, THF-d$_8$, 298 K) δ = 174.6 (NCN), 132.5, 131.5, 131.3, 130.5, 129.8, 129.1, 128.8, 128.7, 126.2 (Ph-*C)*, 55.0 (*C*(CH$_3$)$_3$), 32.3 (C(*C*H$_3$)$_3$). **^{11}B{^{1}H} NMR** (161 MHz, THF-D$_8$, 298 K) δ = -3.2, -7.2, -16.6, -32.6.

<u>Oxidation of **3**</u>

Dimer **3** (50 mg, 0.051 mmol) was added to a Schlenk flask with I_2 crystals (15 mg, 0.059 mmol, 1.16 equiv) and then dissolved in toluene (6 mL). The reaction was stirred overnight (ca. 16 h) and the red color of the solution faded to pale yellow and appeared cloudy. The toluene was removed *in vacuo* and the residue was extracted with 1,2-difluorobenzene (3 + 1 mL) and filtered *via* filter cannula. The solvent was removed *in vacuo*, leaving **2a** as a colorless solid (39.5 mg, 65%).

Compound **4a**

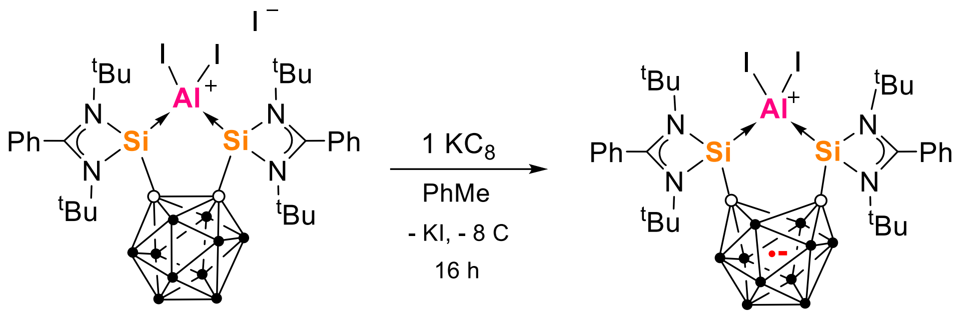

2a **4a** (24%)

Complex **2a** (0.36 g, 0.34 mmol) was mixed with KC_8 (0.115 g, 0.85 mmol, 2.5 equiv) in a Schlenk flask and then suspended in toluene. The suspension was stirred overnight (ca. 16 h) to give a dark brown suspension. The suspension was allowed to settle then cannula filtered to give a dark red solution. The solution was stored at -22 °C for 4 d giving dark brown crystals of **4a** (75 mg, 24%). These crystals were suitable for scXRD analysis. **4a** is NMR silent and was characterized by EPR.

Compound **4b**

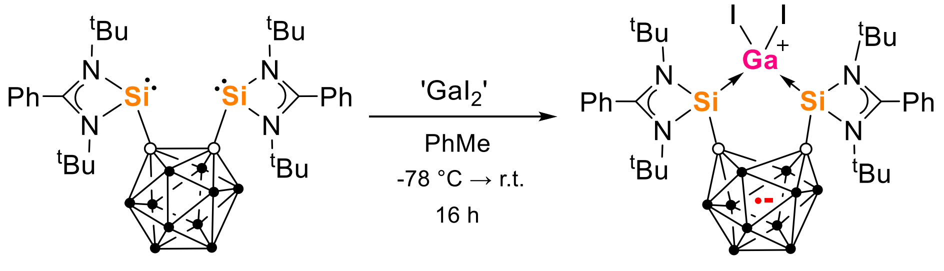

1a **4b** (27%)

Ligand **1a** (250 mg 0.38 mmol), was mixed with 'GaI_2' (170 mg, 0.53 mmol, 1.4 equiv) in a Schlenk flask. The mixture was cooled to -78 °C and toluene (ca. 50 mL) was added with rapid stirring. The reaction flask was allowed to slowly warm to room temperature and stirred overnight (ca. 16 h). On warming, the golden color of the bis(silylene) darkens with the reaction turning into a dark red suspension. The suspension was allowed to settle then cannula filtered to give a dark red-purple solution. The solution was stored at -22 °C overnight giving a mixture of dark red crystals and yellow crystals of **4b** in three crops (combined 102 mg, 27%). Both crystal types were measured by scXRD, with the yellow crystals giving better quality data. **4b** is NMR silent and was characterized by EPR.

HRMS (APCI) *m/z* calcd for $C_{32}H_{56}B_{10}N_4Si_2GaI_2$: 983.2386 [*M*]; found: 983.2381; 1056.2887 [*M*+4(H_2O)]; found: 1056.2959. Elemental analysis calculated for $C_{32}H_{56}B_{10}N_4Si_2GaI_2$: C 39.04, H 5.73, N 5.69; found: C 38.22, H 6.06, N, 5.46.

Compound **5a**

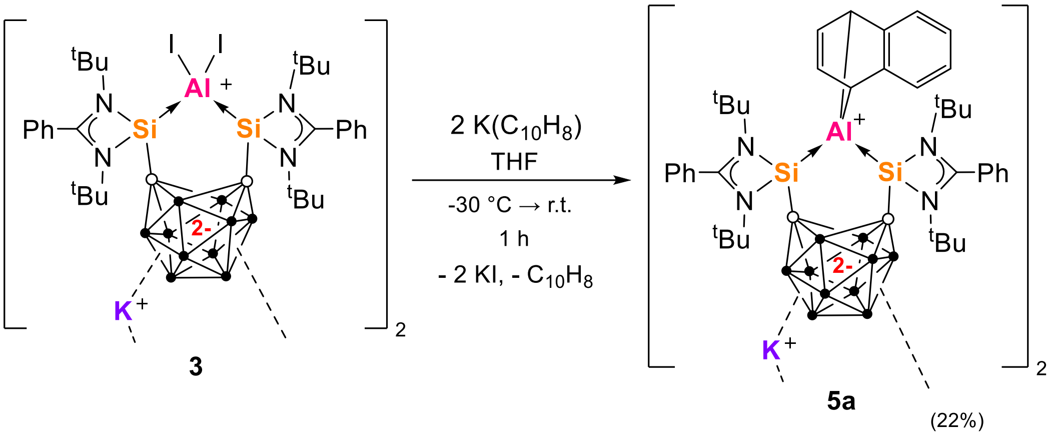

Dimer **3** (0.250 g, 0.127 mmol, 1 equiv) was mixed with K($C_{10}H_8$) (85 mg, 0.508 mmol, 4 equiv) in a Schlenk flask. The mixture was cooled to -30 °C and THF (7 mL) was added with rapid stirring and allowed to warm quickly to room temperature. The mixture was then stirred for 1 h then cannula filtered, and the solvent removed *in vacuo*. The brown residue was extracted with toluene (3 x 30 mL) and filtered via cannula into a new flask. The cloudy orange filtrate was stored at -22 °C for 7 d, yielding **5a** as orange crystals (47 mg, 22%). These crystals could not be measured by scXRD.

^{1}H NMR (500 MHz, THF-d_8, 298 K, ppm) δ = 7.56 (d, $^3J_{HH}$ = 7.6 Hz, 1H, Ph*H*), 7.49 (m, 4H, Ph*H*), 7.42 (m, 2H, Ph*H*), 7.35 (td, $^3J_{HH}$ = 7.3 Hz, $^4J_{HH}$ = 1.1 Hz, 1H, Ph*H*), 7.26 (d, $^3J_{HH}$ = 7.6 Hz, 1H, Ph*H*), 7.10 (d, $^3J_{HH}$ = 7.4 Hz, 1fH, Ph*H*), 6.93 (dd, $^3J_{HH}$ = 5.3 Hz, $^4J_{HH}$ = 3.2 Hz, 2 H, Naph-*H*), 6.80 (dd, $^3J_{HH}$ = 5.3 Hz, $^4J_{HH}$ = 3.2 Hz, 2 H, Naph-*H*), 6.38 (dd, $^3J_{HH}$ = 4.5 Hz, $^4J_{HH}$ = 3.2 Hz, 2 H, C=C*H*), 3.04 (m, 2 H, AlC*H*), 1.28 (s, 18 H, C(C*H*$_3$)$_3$), 1.09 (s, 18 H, C(C*H*$_3$)$_3$). **^{13}C{^{1}H} NMR** (125 MHz, THF-d_8, 298 K, ppm) δ = 170.8 (N*C*N), 170.6 (N*C*N), 149.8 (Naph-*C*), 133.7, 133.5, 132.2, 131.2, 131.1, 130.8, 129.8, 129.4, 129.1, 128.8, 128.7, 128.1 (Ph-*C), 126.2 (H*C*=*C*H), 121.7 (Naph-C*H*), 121.4 (Naph-*C*H), 54.5 (*C*(CH$_3$)$_3$), 54.1 (*C*(CH$_3$)$_3$), 48.2 (AlCH), 32.4 (C(*C*H$_3$)$_3$), 31.8 (C(*C*H$_3$)$_3$). **^{11}B NMR** (161 MHz, THF-D$_8$, 298 K, ppm) δ = 10.9 (br.), -1.9 (d, J_{BH} = 96.7 Hz), -15.1 (s), -21.2 (d, J_{BH} = 107.7 Hz). **^{1}H,^{29}Si-HMQC NMR** (500 MHz, THF-D$_8$, 298 K, J_{SiH} = 3z Hz, NUS 50%, ppm) δ_{Si} = 30.7, 40.4.

Compound **5b**

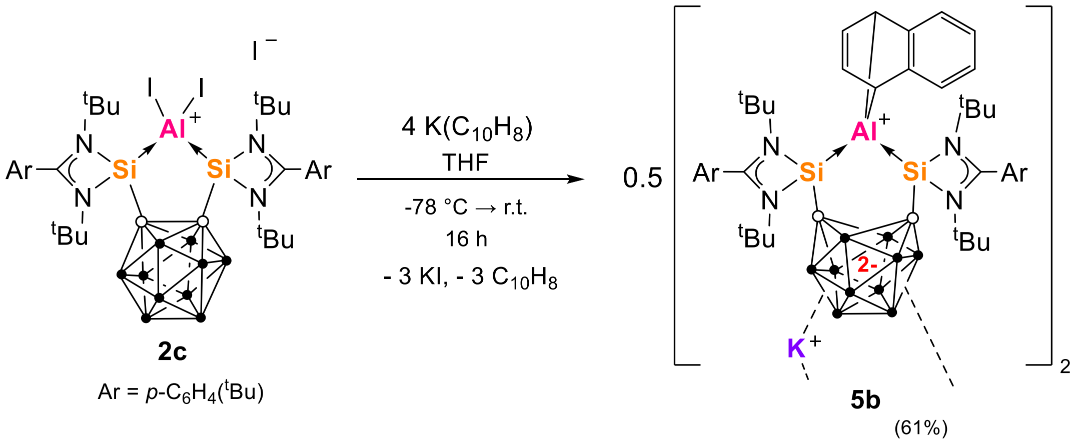

2c (300 mg, 0.252 mmol, 1 equiv) was mixed with $K(C_{10}H_8)$ (170 mg, 1.02 mmol, 4 equiv) in a Schlenk flask in the glovebox. The mixture was cooled to -78 °C and THF (8 mL) was added with rapid stirring and allowed to warm quickly to room temperature, turning dark brown rapidly. The mixture was left to stir for 16 h then the solvent removed *in vacuo*. The brown residue was extracted with Et_2O (x mL) and stirred for 5 min. The mixture was allowed to settle then filtered via cannula into a new flask. The brown filtrate was concentrated and then stored at -22 °C for 3 d, yielding red-orange crystals. The crystals were separated from the supernatant and washed with 2 x 1 mL Et_2O before drying *in vacuo* to give **5b** as an orange solid (161 mg, 61%). Crystals of **5b** grown from Et_2O solutions were suitable for scXRD analysis.

^{1}H NMR (500 MHz, C_6D_6, 298 K, ppm) δ = 7.48 (dd, $^3J_{HH}$ = 5.2 Hz, $^4J_{HH}$ = 3.2 Hz, 2H, Ar*H*), 7.39 (dd, $^3J_{HH}$ = 8.2 Hz, $^4J_{HH}$ = 1.8 Hz, 1H, Ar*H*), 7.33 (d, $^3J_{HH}$ = 8.1 Hz, 1H, Ar*H*), 7.29 (dd, $^3J_{HH}$ = 8.2 Hz, $^4J_{HH}$ = 1.4 Hz, 1H, Ar*H*), 7.27 – 7.11 (m, 6H, Ar*H*), 7.07 (d, $^3J_{HH}$ = 8.1 Hz, 1H, Ar*H*), 6.94 (t, $^3J_{HH}$ = 3.8 Hz, 2H, C=C*H*), 3.62 (t, $^3J_{HH}$ = 3.8 Hz, 2H, AlC*H*), 1.45 (s, 18H, NC(CH_3)$_3$), 1.35 (s, 18H, NC(CH_3)$_3$), 1.13 (s, 18H, Ar-C(CH_3)$_3$). **^{13}C{^{1}H} NMR** (125 MHz, C_6D_6, 298 K, ppm) δ = 171.7 (N*C*N), 171.6 (N*C*N), 153.8, 153.6, 149.1, 134.1, 131.0, 129.8, 129.5, 129.0, 128.9, 128.7, 128.6, 128.2, 128.0, 126.1, 125.1, 125.0, 124.9, 124.8, 124.5, 121.9, 121.6 (Ar*C, C*=*C*), 67.9 (*C*Si), 54.0 (N*C*(CH_3)$_3$), 53.7 (N*C*(CH_3)$_3$), 47.7 (Al*C*H), 34.8 (Ar-*C*(CH_3)$_3$, 34.8 (Ar-*C*(CH_3)$_3$, 32.2 (NC(*C*H_3)$_3$), 32.0t (NC(*C*H_3)$_3$), 31.2 (Ar-C(*C*H_3)$_3$). **^{11}B NMR** (161 MHz, C_6D_6, 298 K, ppm) δ = 0.3 (br.), -6.1 (br.), -15.4 (br.), -30.0 (br.). **^{29}Si NMR** (99 MHz, C_6D_6, 298 K, ppm) δ = 29.4, 39.1. **HRMS** (ESI) *m/z* calcd for fragment $C_{31}H_{60}B_{10}N_2SiAlO_5$: 705.5241 [*M*–$K^+$–($C_{19}H_{31}N_2Si$)+5($H_2O$)]; found: 705.4322.

Attempts to trap fleeting low valent Al species

a) Dropwise addition of crystalline [K($C_{10}H_8$)(THF)] (4 molar equivs) dissolved in THF to a suspension of 1 molar equiv **2c** and 1 equiv ^{Me}cAAC-5 cooled to -78 °C gave a dark brown mixture. On warming it lightened to a golden orange color after stirring 1 h at r.t.. The solvent was removed in vacuo and the residue extracted with Et_2O, but no crystalline material grew after 3 weeks. ^{1}H NMR aliquots showed naphthalene as the major product and a complicated mixture.

b) In an attempt to access a radical product directly, 1 equiv [K($C_{10}H_8$)(THF)] dissolved in THF was added dropwise to a solution of 0.5 molar equiv of dimer **3** with and 1 equiv ^{Me}cAAC-5 in THF cooled to -78 °C. This resulted in a dark red filtrate. No products could be crystallized from THF alone or with layering of Et_2O.

c) Reduction of the BPh_4 salt **2d** with K/KI (approx. 5 equiv K, 5% w/w) in Et_2O in the presence of one equiv ^{Me}cAAC-5 led to the formation of very dark blue mixtures after a few hours of stirring. This was promising as unsaturated silylene- or cAAC-Al species (e.g. **III**, Figure 1) have been reported to give solutions with intense colors, such as dark-violet, -blue and -green.[15–17] Using toluene as the solvent instead, a dark blue mixture was only obtained in 48 hours of stirring. The blue color was not observed when using **2c** as a reactant (instead of **2d**) or without the addition of cAAC. While some small blue crystals could be grown from Et_2O/hexane solution, they decomposed during attempted measurement, deforming and changing color. All other attempts to isolate the dark blue compound from Et_2O, toluene, benzene/hexane and iPr_2O as single-crystals suitable for scXRD failed.

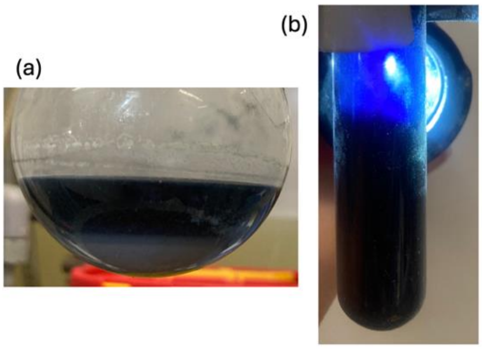

Figure S1. Color of the resulting mixture from the reaction of **2d** with K/KI and ^{Me}cAAC-5. (a) reaction mixture allowed to settle (b) filtered reaction mixture held in front of a torch.

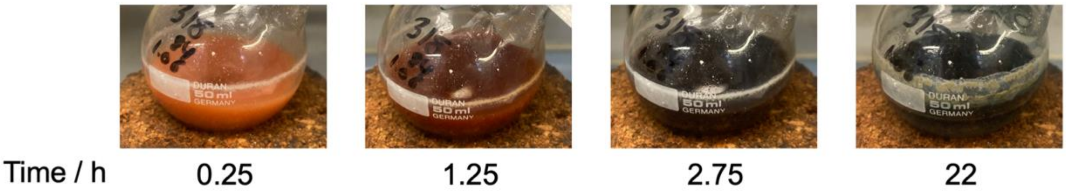

Figure S2. Color changes at the different time points for the reaction of **2d** with K/KI and ^{Me}cAAC-5.

A3 NMR Spectra

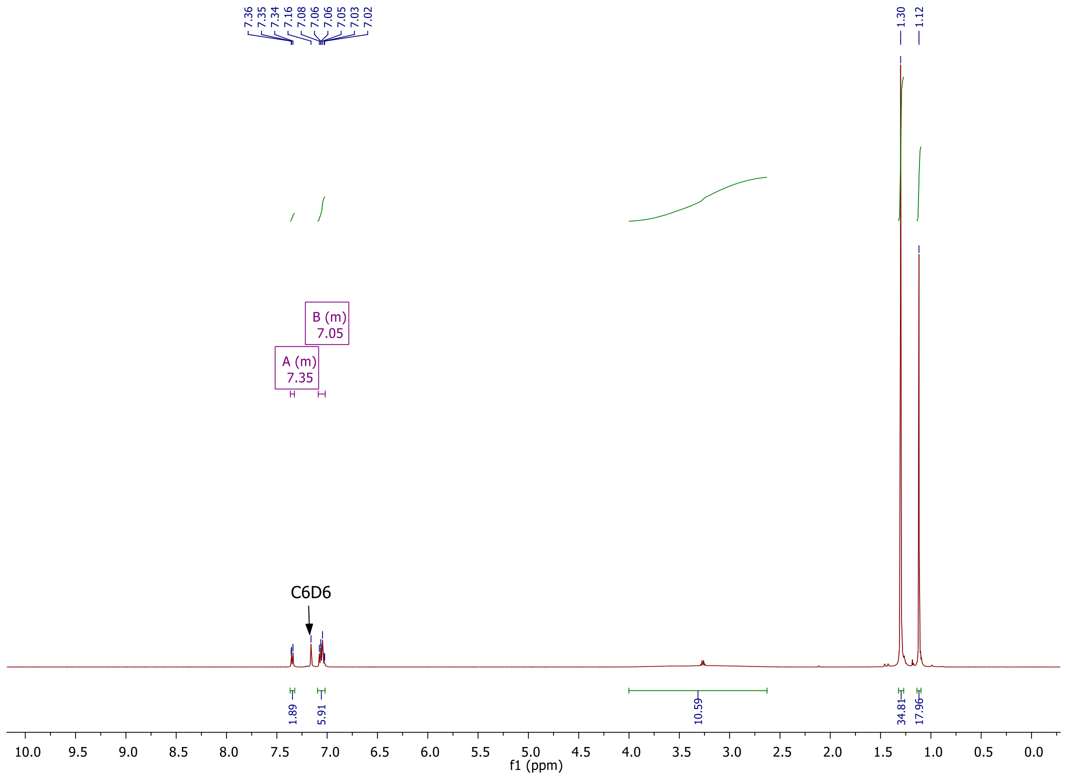

Figure S3 ^{1}H NMR spectrum of **1b** in C_6D_6 under 1 bar N_2 at 298 K.

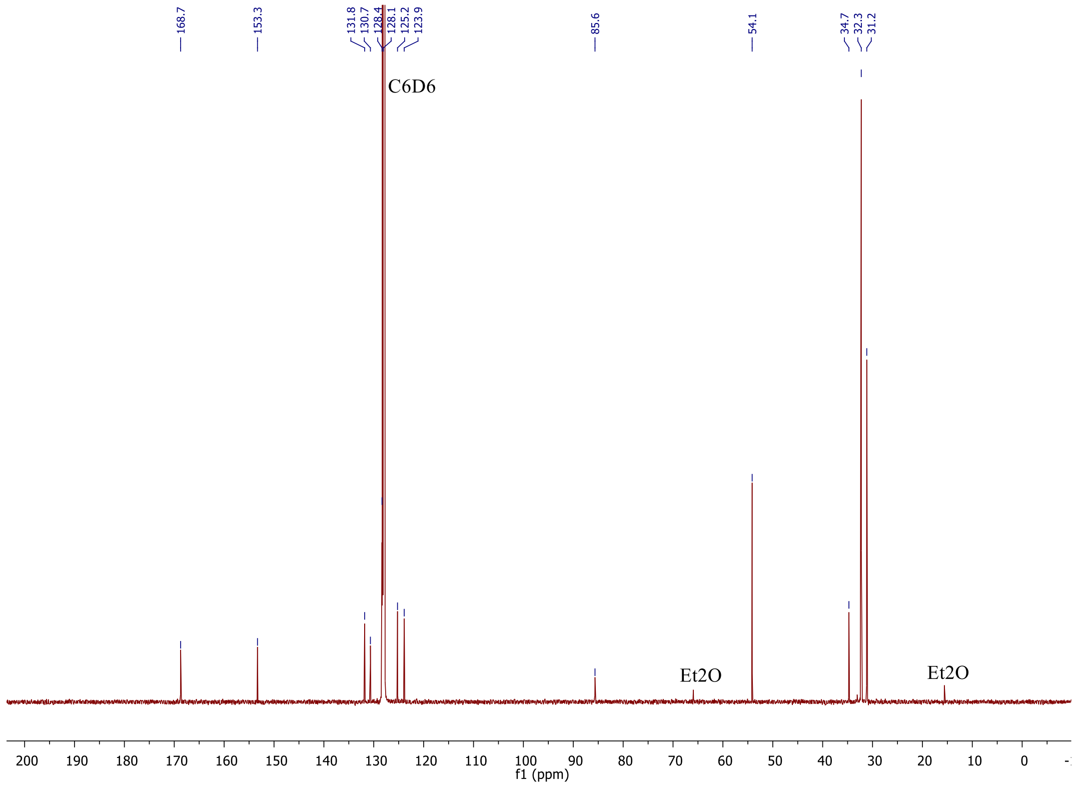

Figure S4 $^{13}C\{^{1}H\}$ NMR spectrum of **1b** in C_6D_6 under 1 bar N_2 at 298 K.

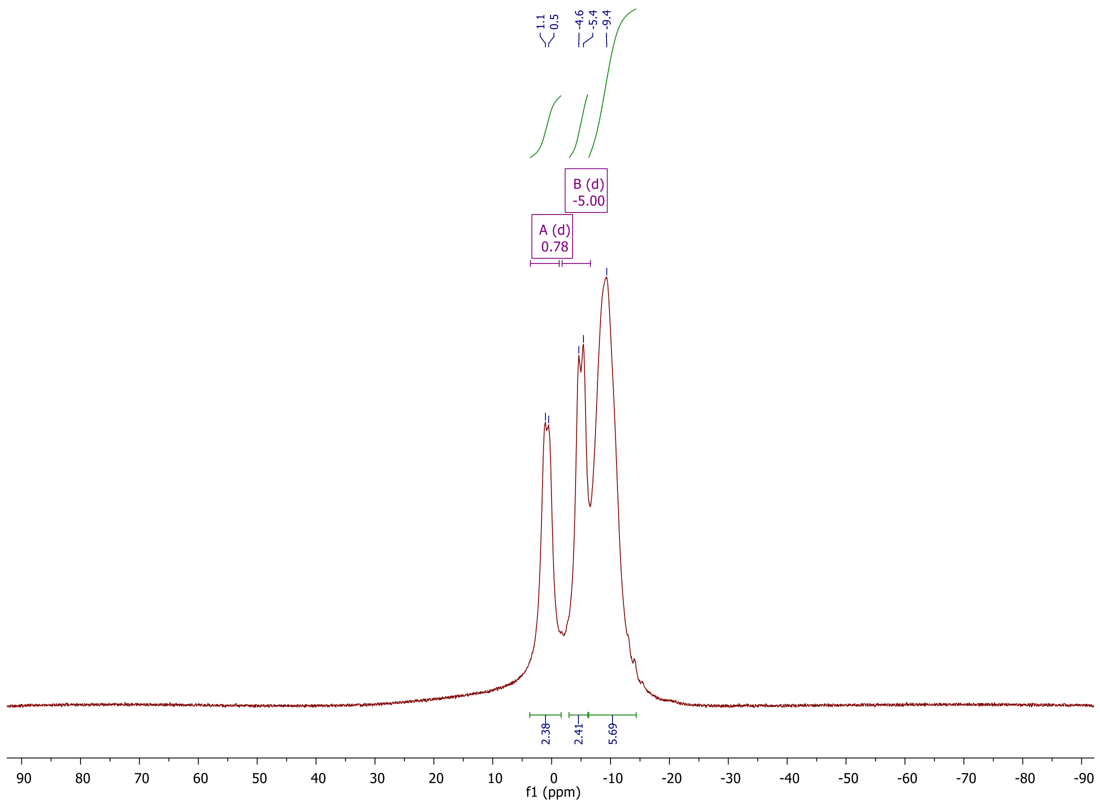

Figure S5 ^{11}B NMR spectrum of **1b** in C_6D_6 under 1 bar N_2 at 298 K.

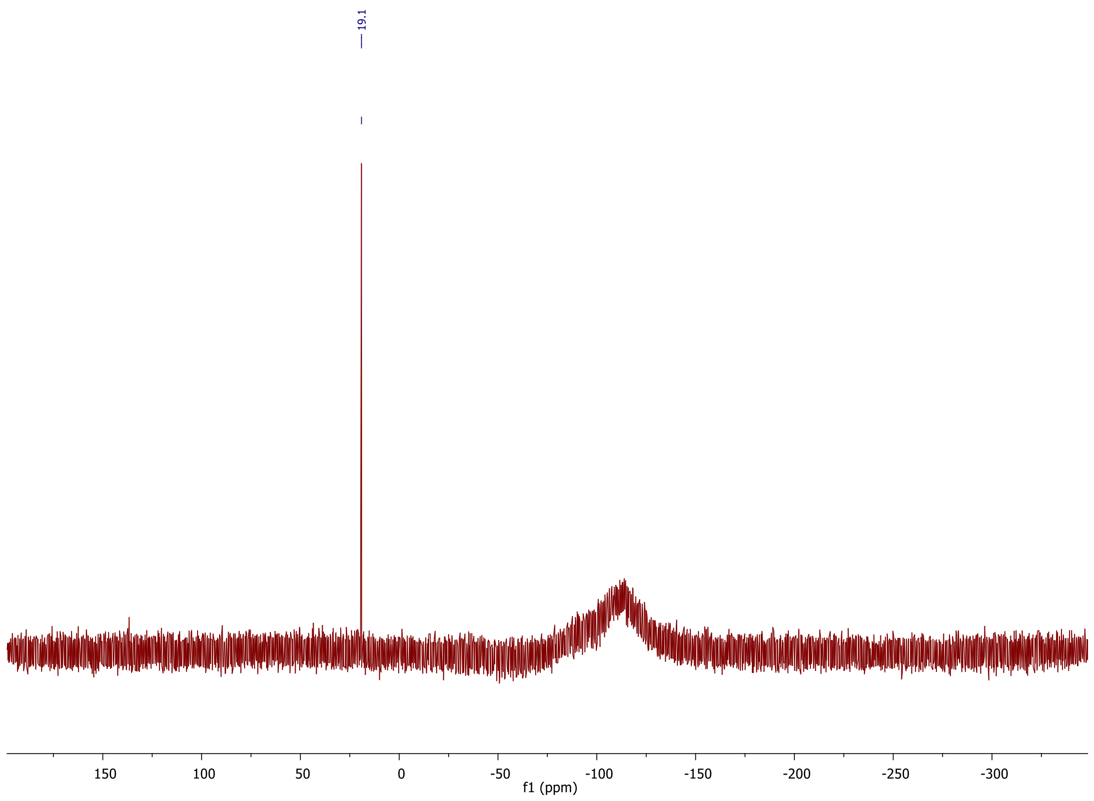

Figure S6 $^{29}Si\{^1H\}$ NMR spectrum of **1b** in C_6D_6 under 1 bar N_2 at 298 K.

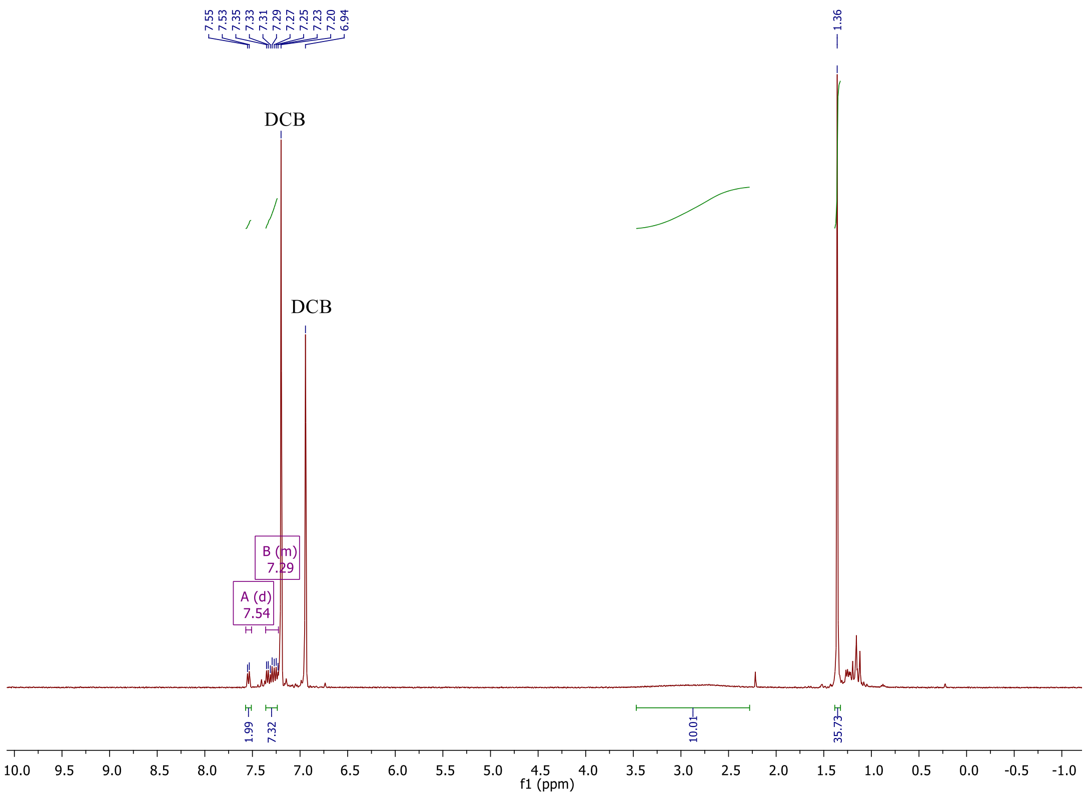

Figure S7 ^{1}H NMR spectrum of **2a** in *o*-$C_6D_4Cl_2$ under 1 bar N_2 at 298 K.

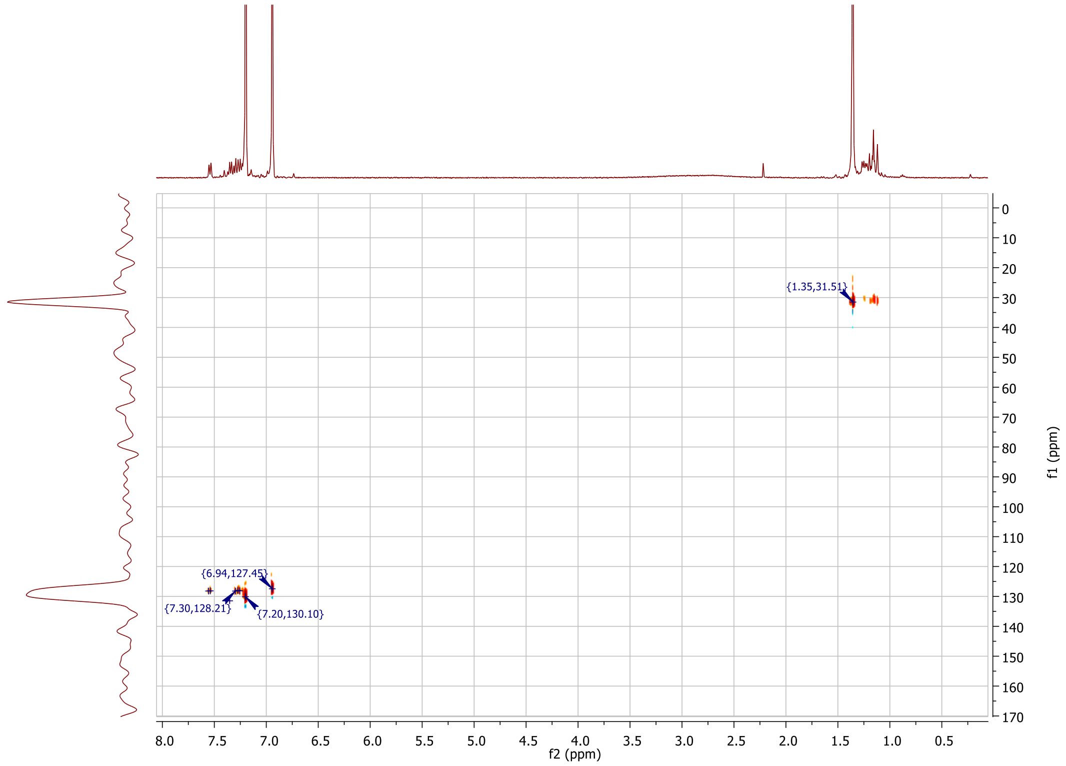

Figure S8 ^{1}H,^{13}C-HSQC NMR spectrum of **2a** in *o*-$C_6D_4Cl_2$ under 1 bar N_2 at 298 K.

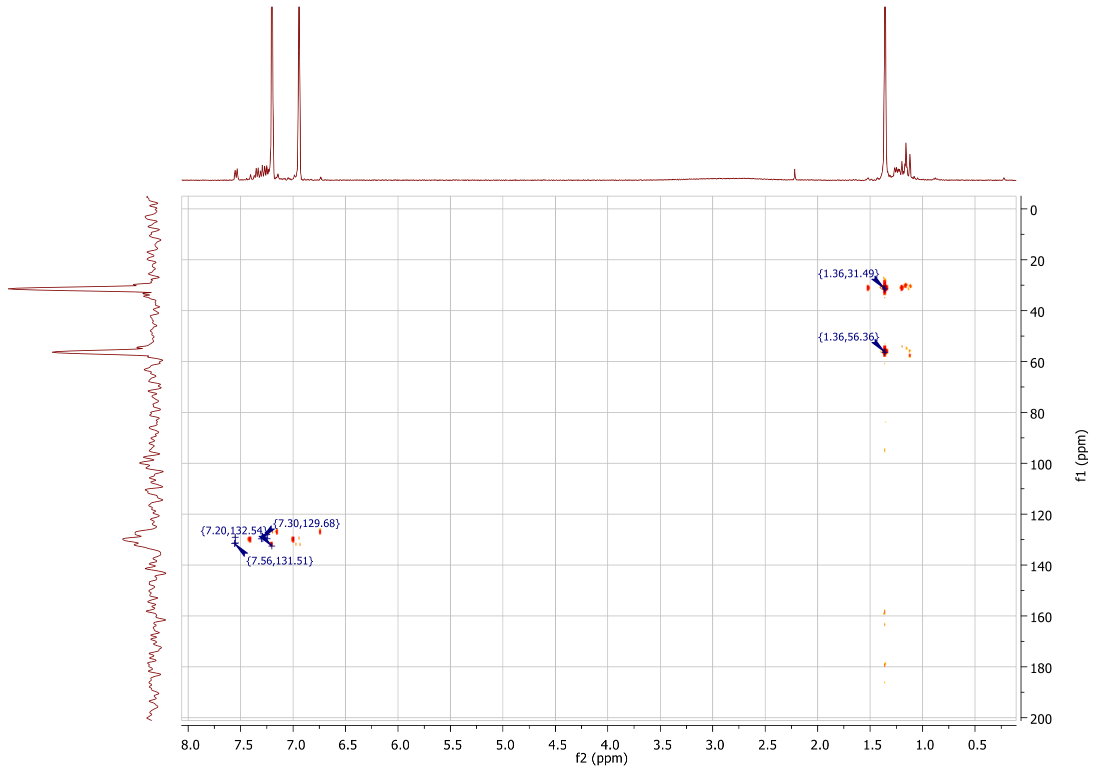

Figure S9 $^{1}H,^{13}C$-HMBC NMR spectrum of **2a** in *o*-$C_6D_4Cl_2$ under 1 bar N_2 at 298 K.

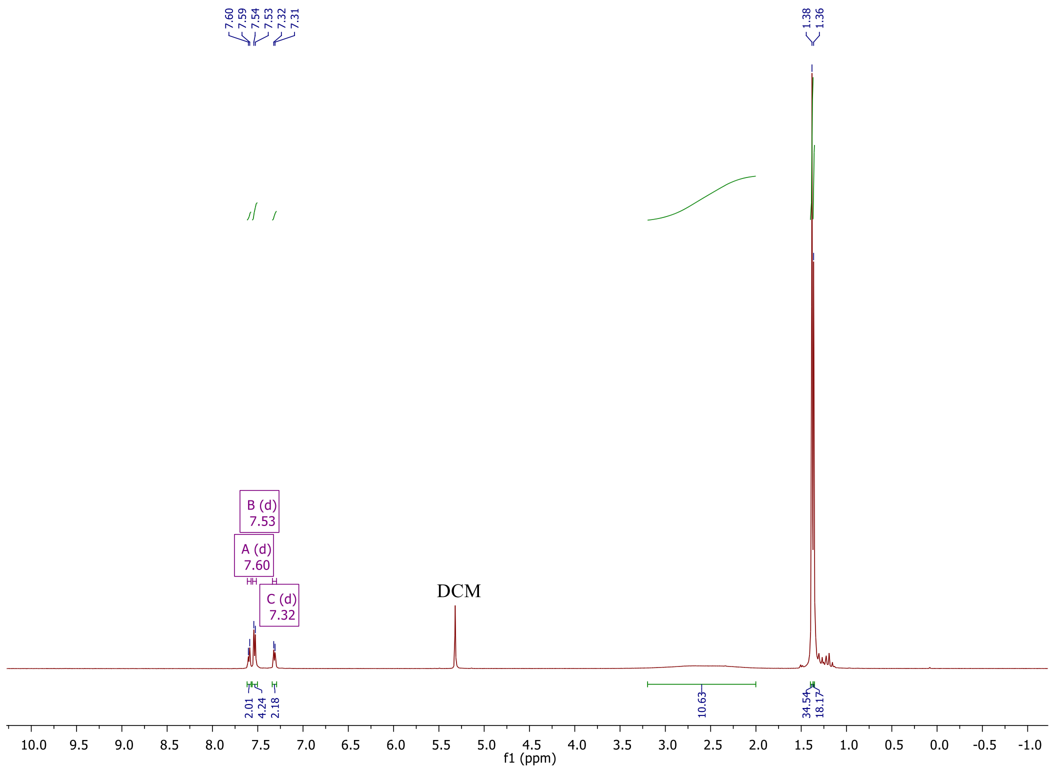

Figure S10 ^{1}H NMR spectrum of **2c** in CD_2Cl_2 under 1 bar N_2 at 298 K.

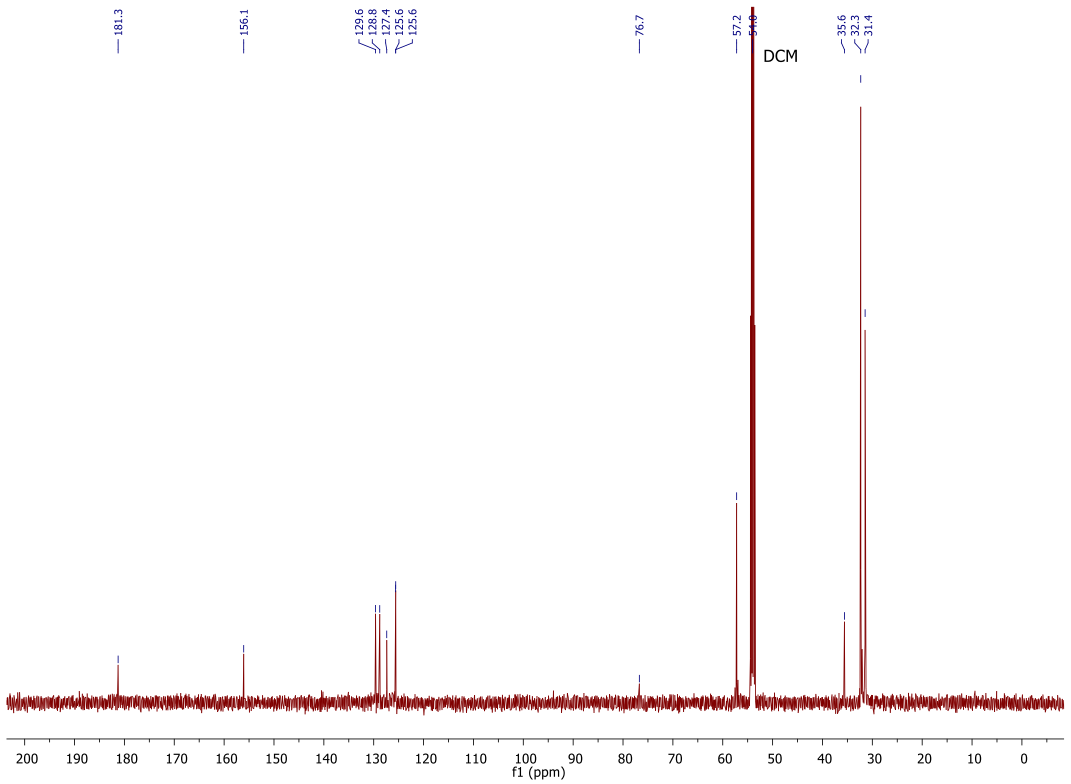

Figure S11 $^{13}C\{^{1}H\}$ NMR spectrum of **2c** in CD_2Cl_2 under 1 bar N_2 at 298 K.

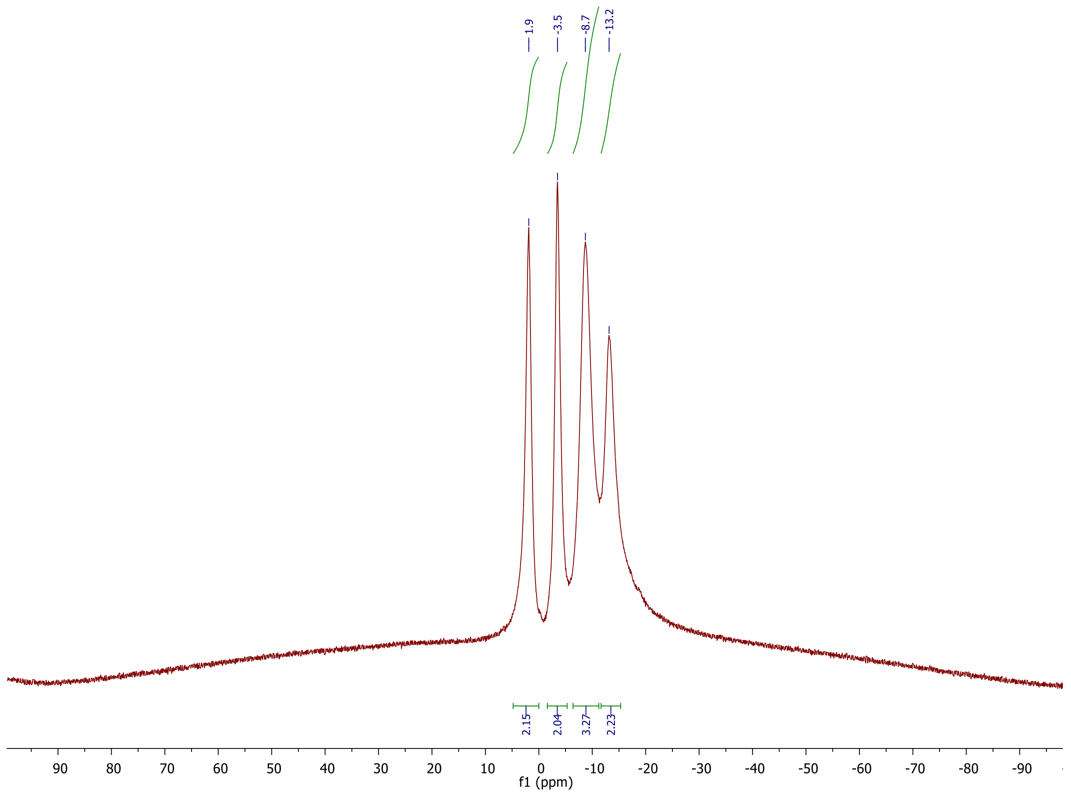

Figure S12 $^{11}B\{^{1}H\}$ NMR spectrum of **2c** in CD_2Cl_2 under 1 bar N_2 at 298 K.

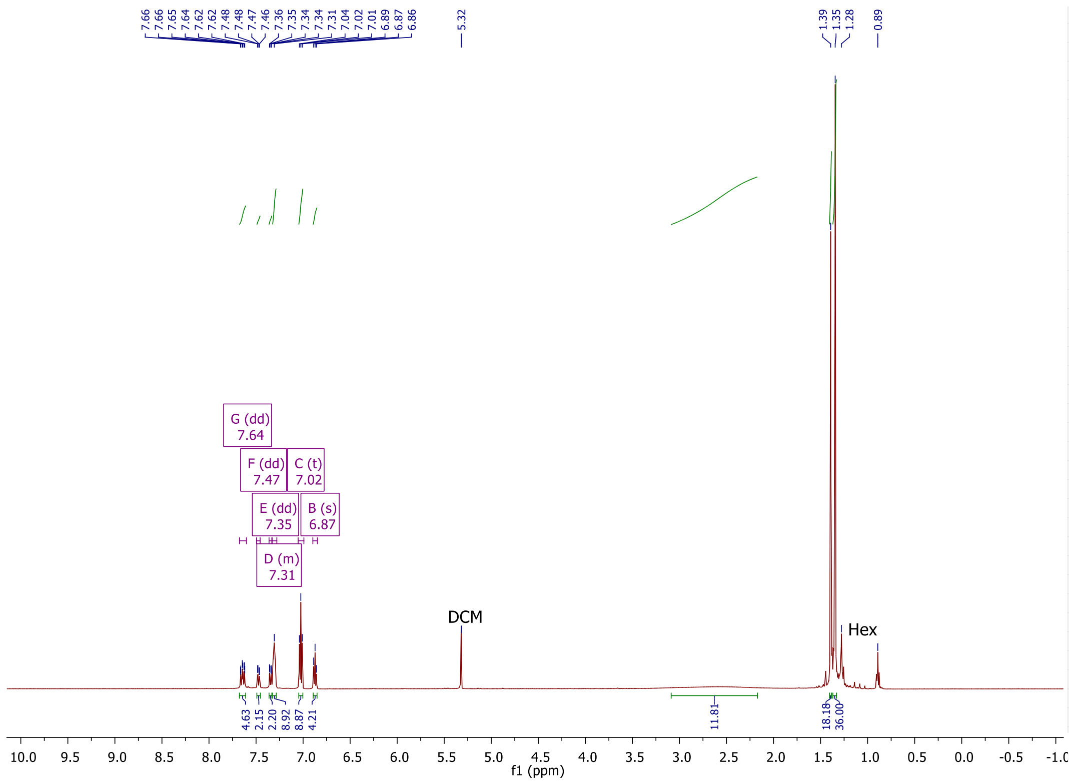

Figure S13 ^{1}H NMR spectrum of **2d** in CD_2Cl_2 under 1 bar N_2 at 298 K.

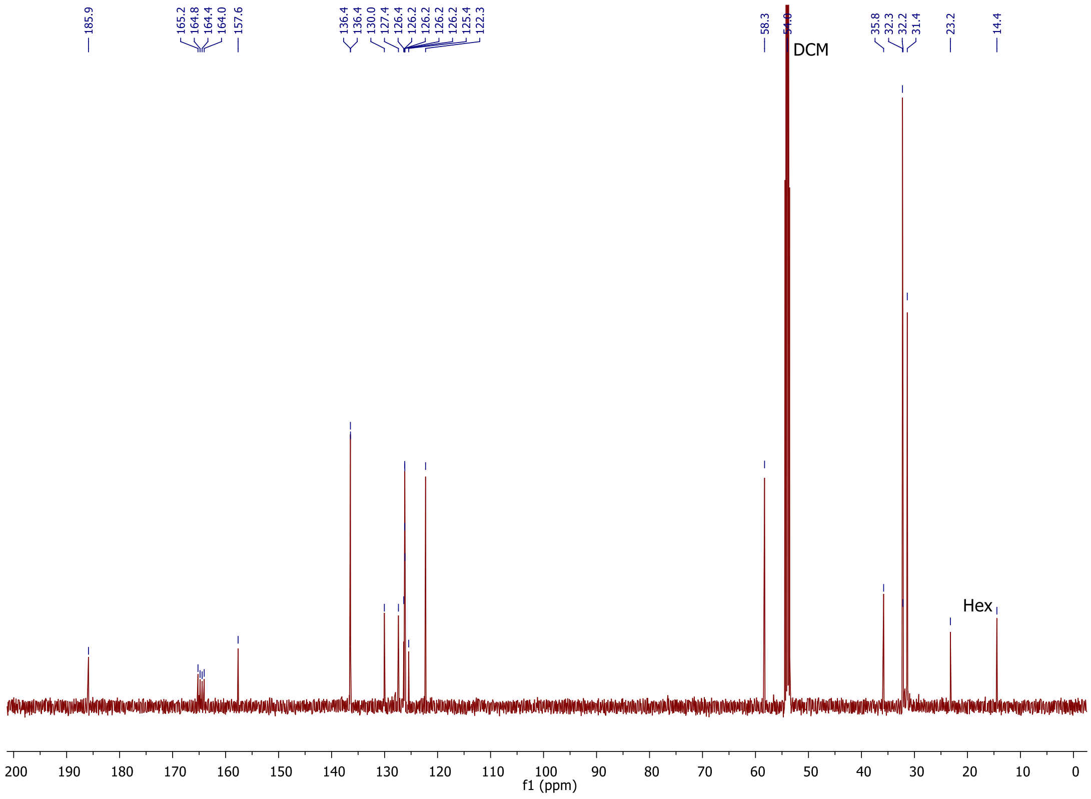

Figure S14 $^{13}C\{^{1}H\}$ NMR spectrum of **2d** in CD_2Cl_2 under 1 bar N_2 at 298 K.

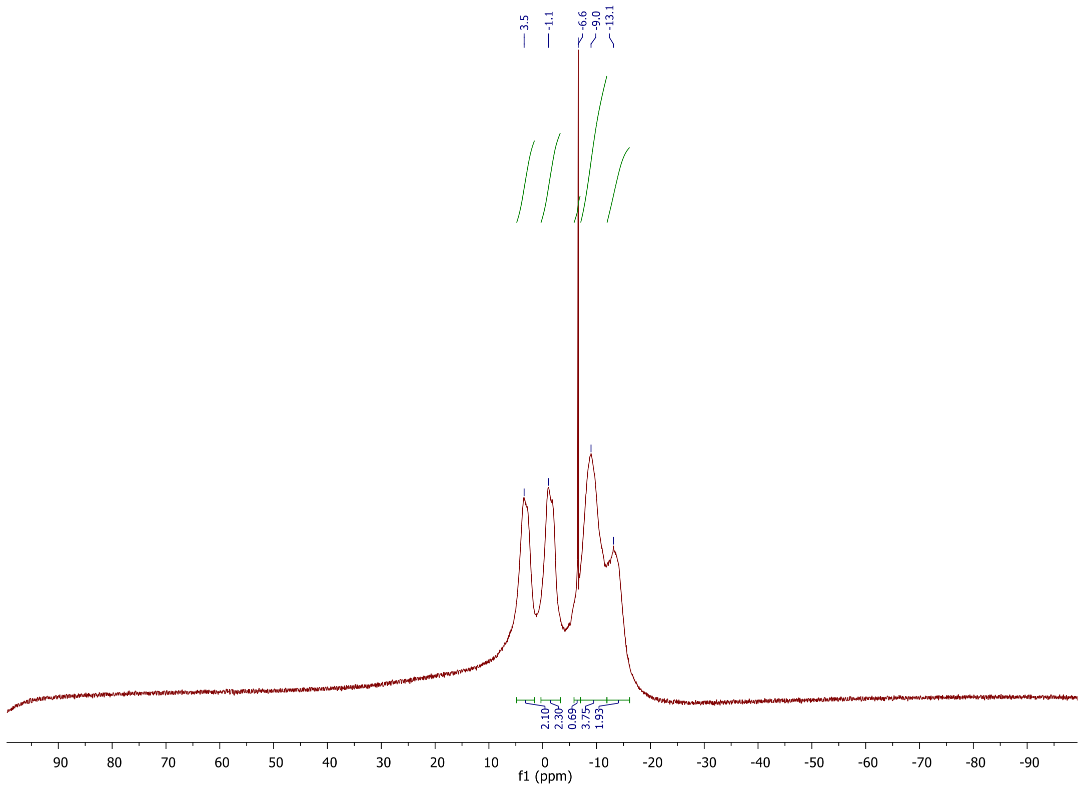

Figure S15 ^{11}B NMR spectrum of **2d** in CD_2Cl_2 under 1 bar N_2 at 298 K.

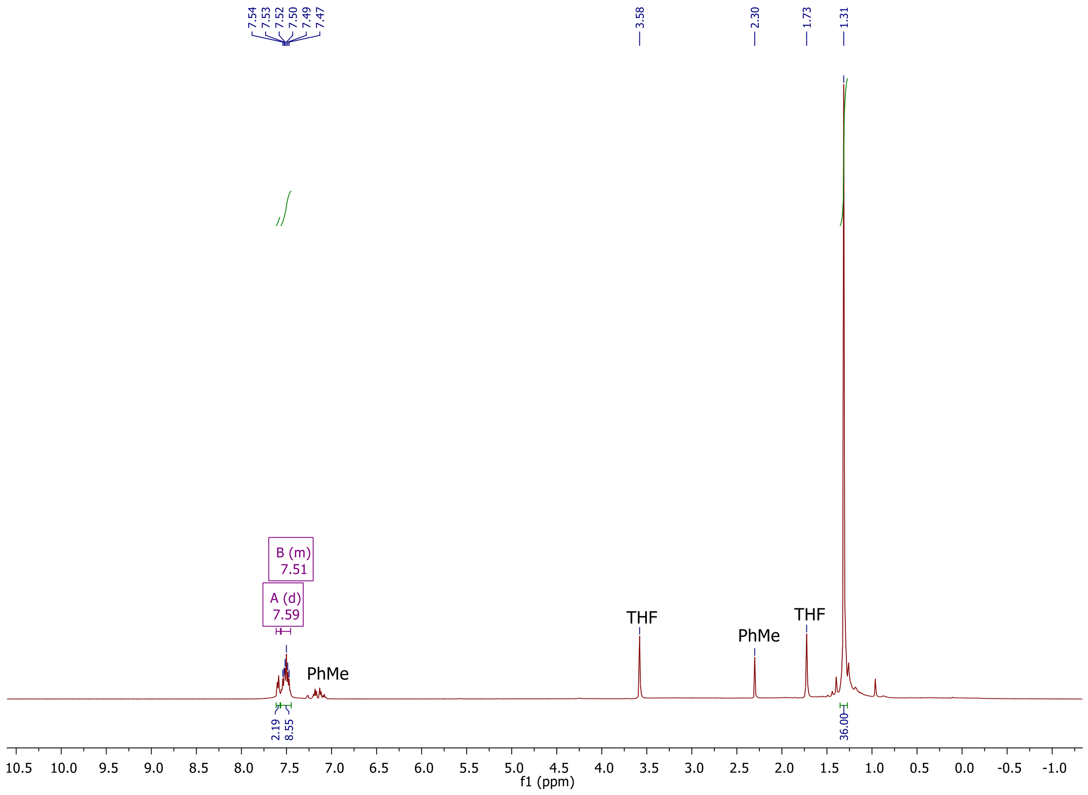

Figure S16 ^{1}H NMR spectrum of **3** in THF-D_8 under 1 bar N_2 at 298 K.

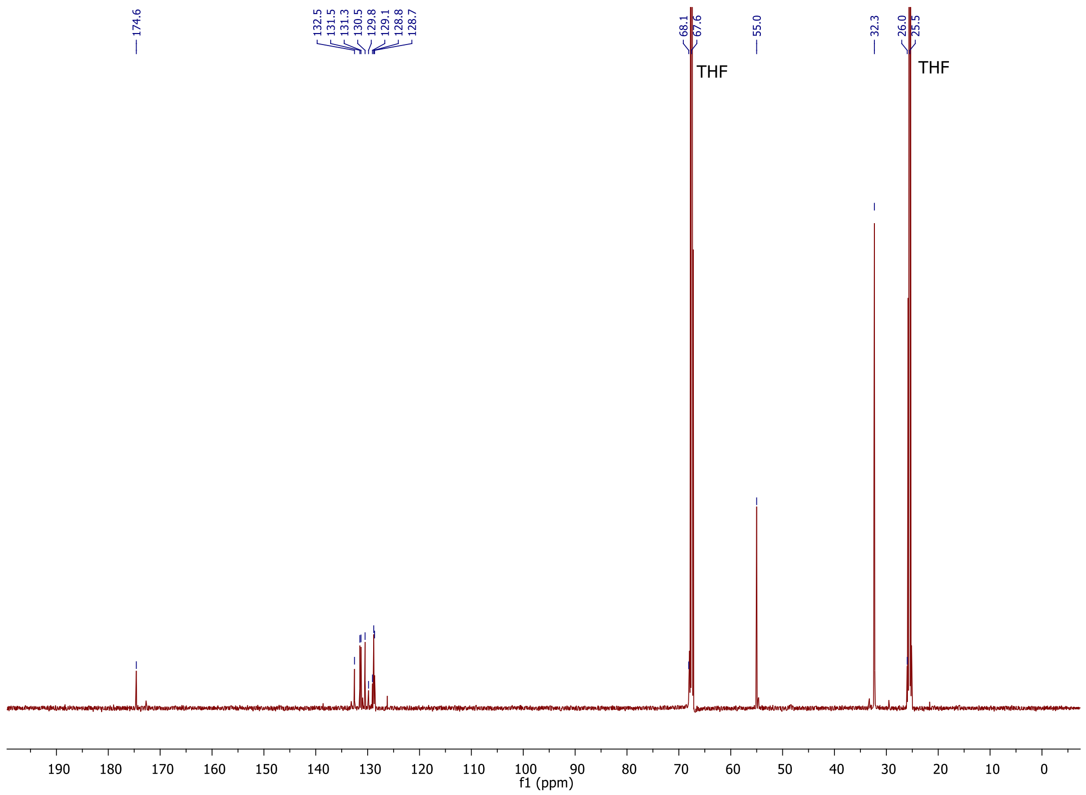

Figure S17 $^{13}C\{^1H\}$ NMR spectrum of **3** in THF-D_8 under 1 bar N_2 at 298 K.

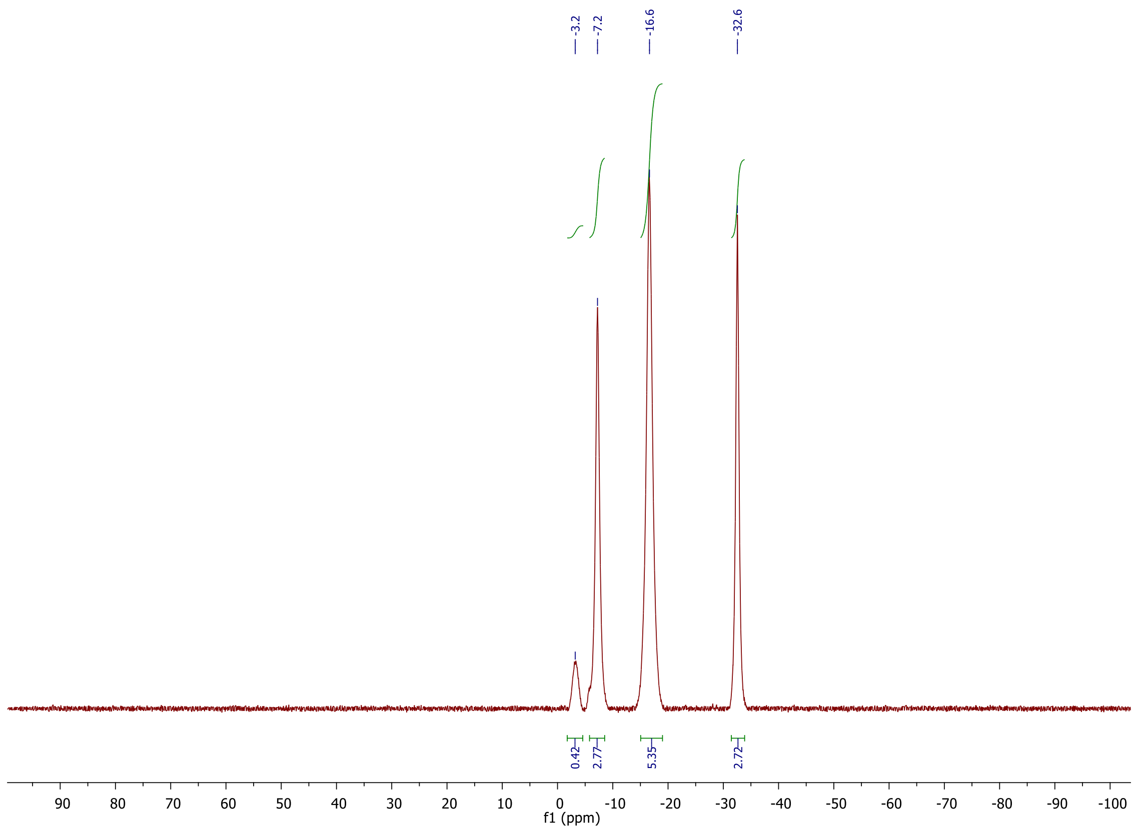

Figure S18 $^{11}B\{^1H\}$ NMR spectrum of **3** in THF-D_8 under 1 bar N_2 at 298 K.

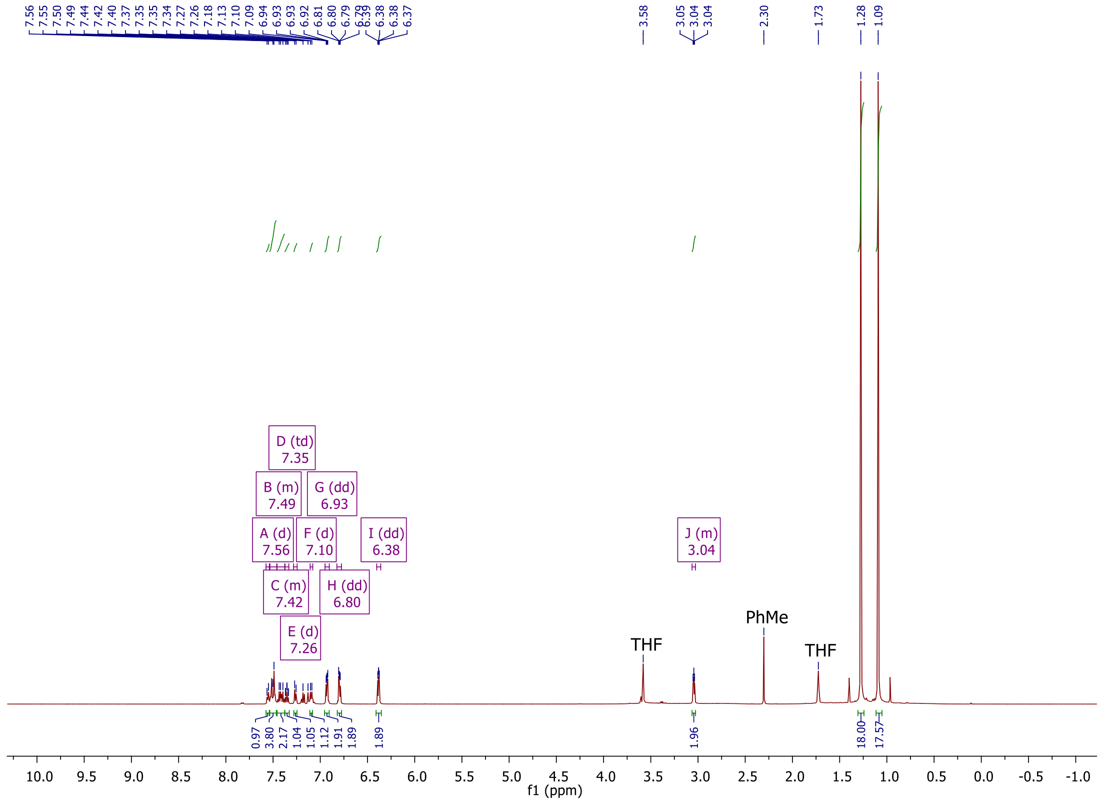

Figure S19 ^{1}H NMR spectrum of **5a** in THF-D_8 under 1 bar N_2 at 298 K.

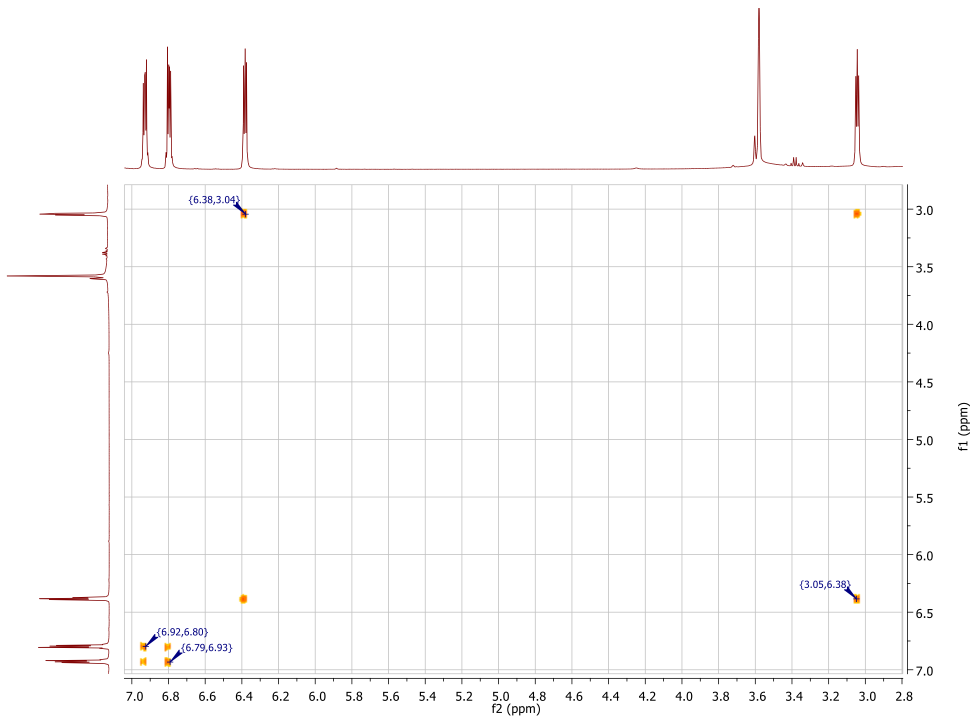

Figure S20 ^{1}H,^{1}H-COSY NMR spectrum of **5a** in THF-D_8 under 1 bar N_2 at 298 K.

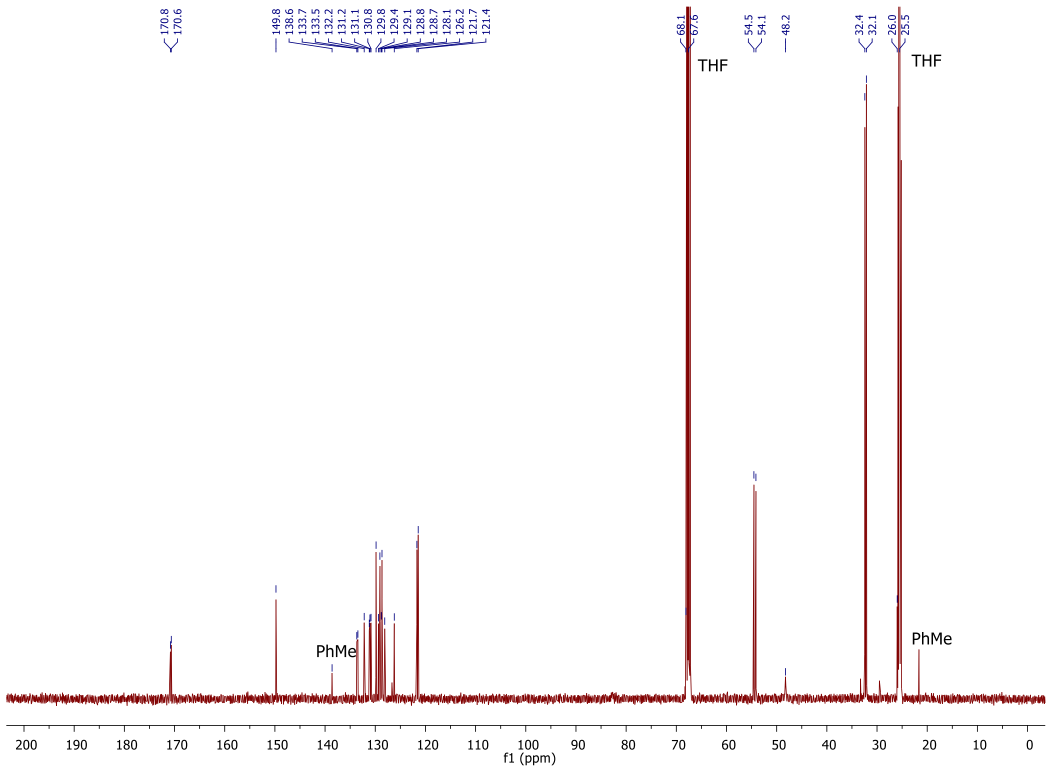

Figure S21 $^{13}C\{^{1}H\}$ NMR spectrum of **5a** in THF-D$_8$ under 1 bar N_2 at 298 K.

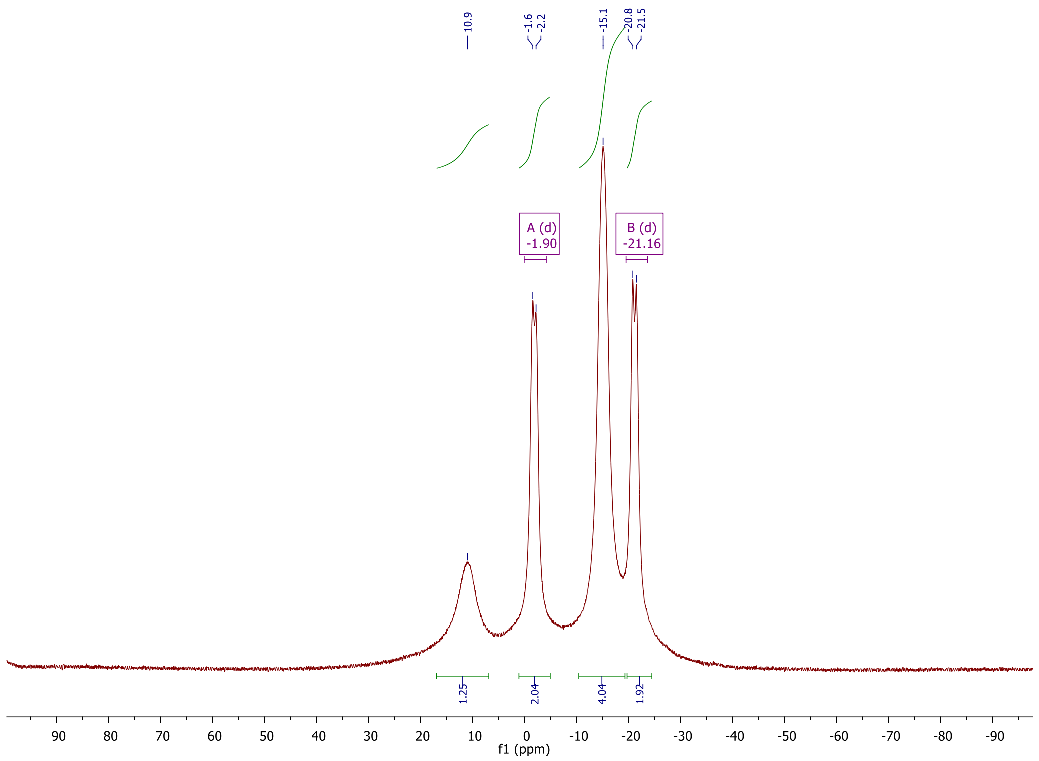

Figure S22 ^{11}B NMR spectrum of **5a** in THF-D$_8$ under 1 bar N_2 at 298 K.

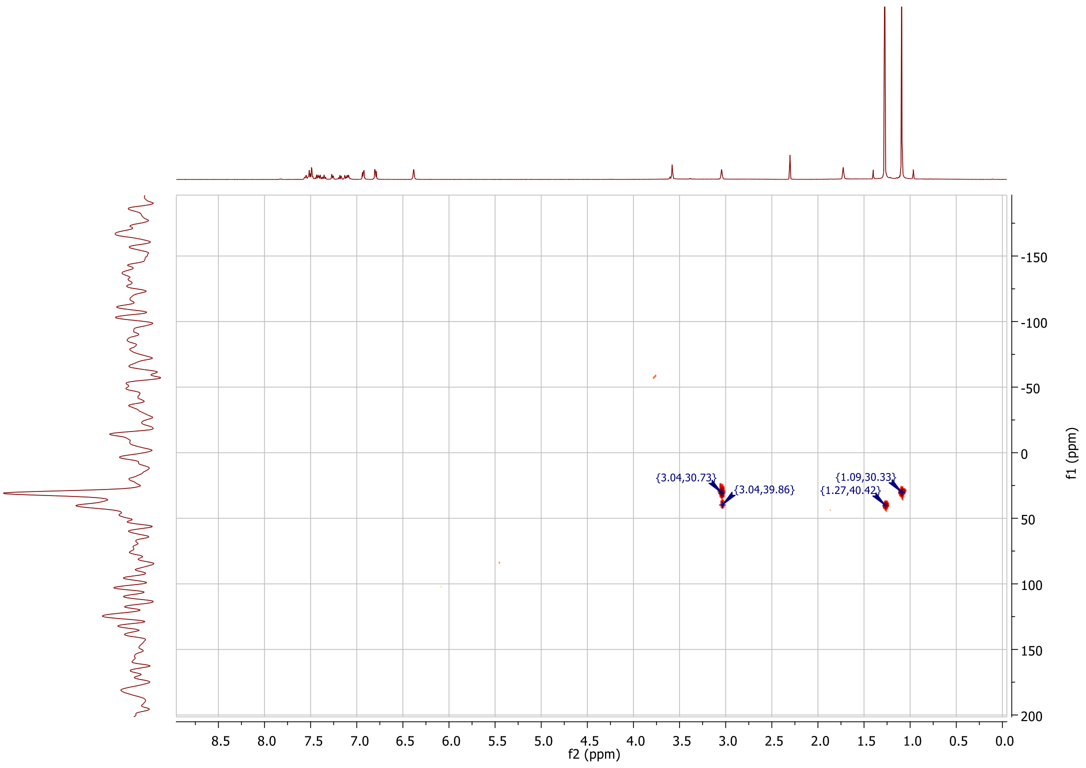

Figure S23 ^{1}H,^{29}Si-HMQC NMR spectrum of **5a** in THF-D$_8$ under 1 bar N$_2$ at 298 K (NUS 50%, J_{HSi} = 3 Hz).

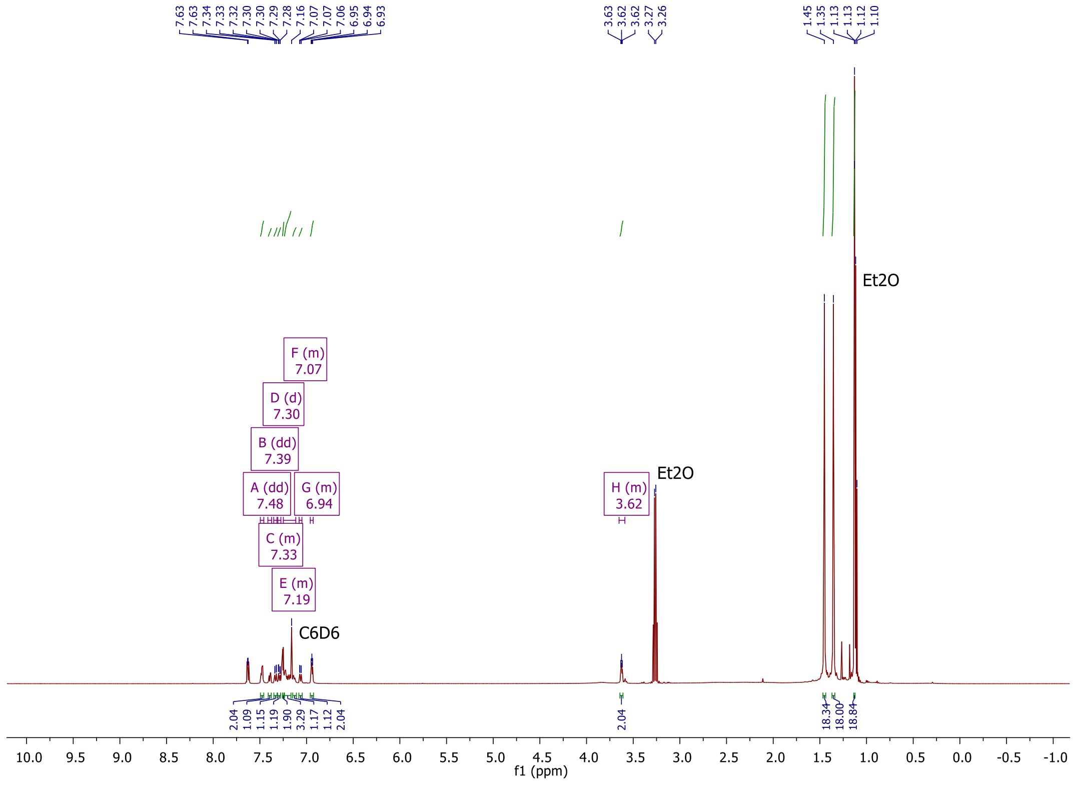

Figure S24 ^{1}H NMR spectrum of **5b** in C$_6$D$_6$ under 1 bar N$_2$ at 298 K.

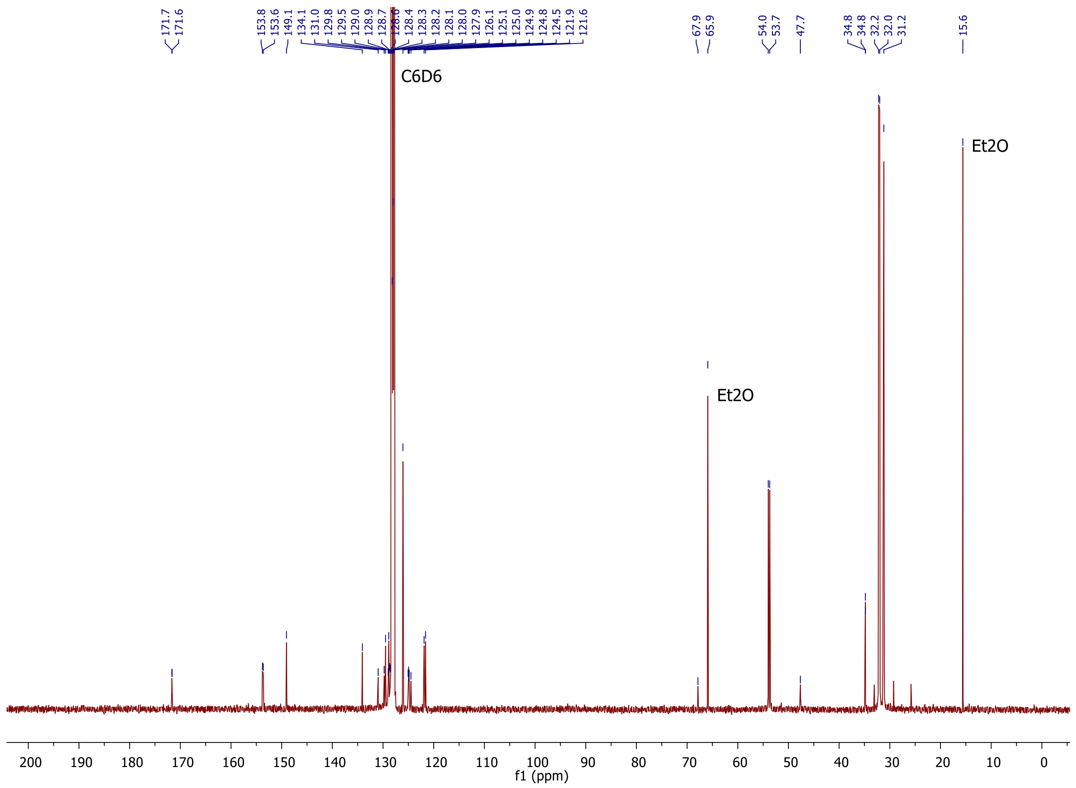

Figure S25 $^{13}C\{^{1}H\}$ NMR spectrum of **5b** in C_6D_6 under 1 bar N_2 at 298 K.

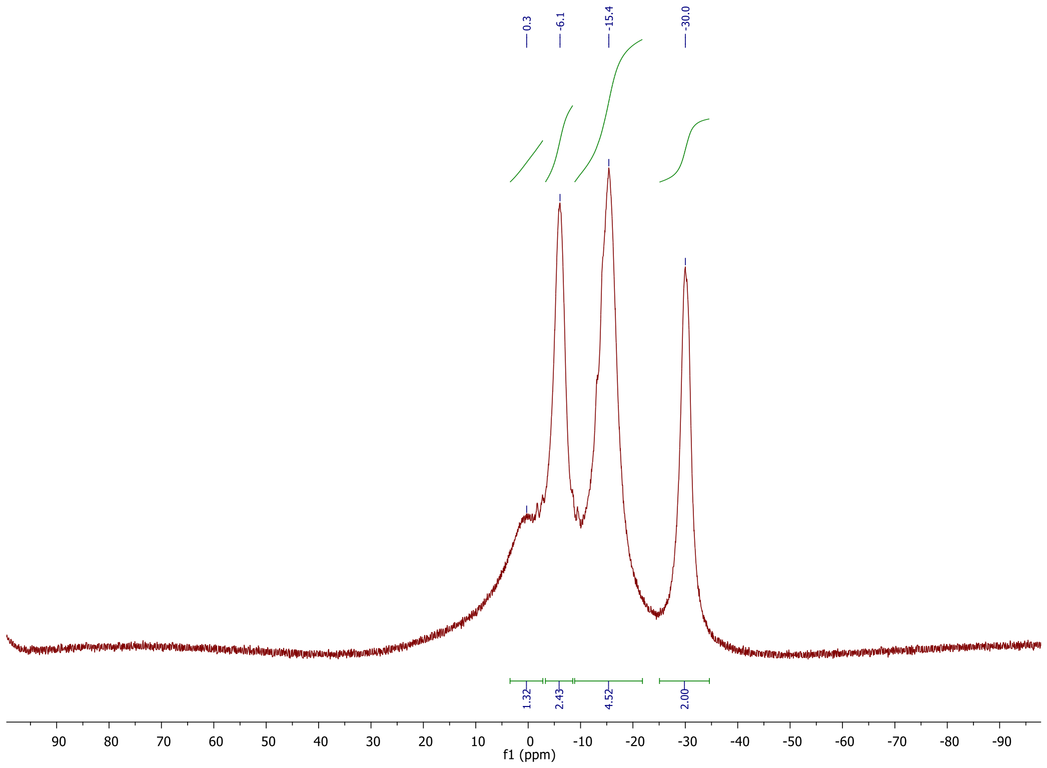

Figure S26 ^{11}B NMR spectrum of **5b** in C_6D_6 under 1 bar N_2 at 298 K.

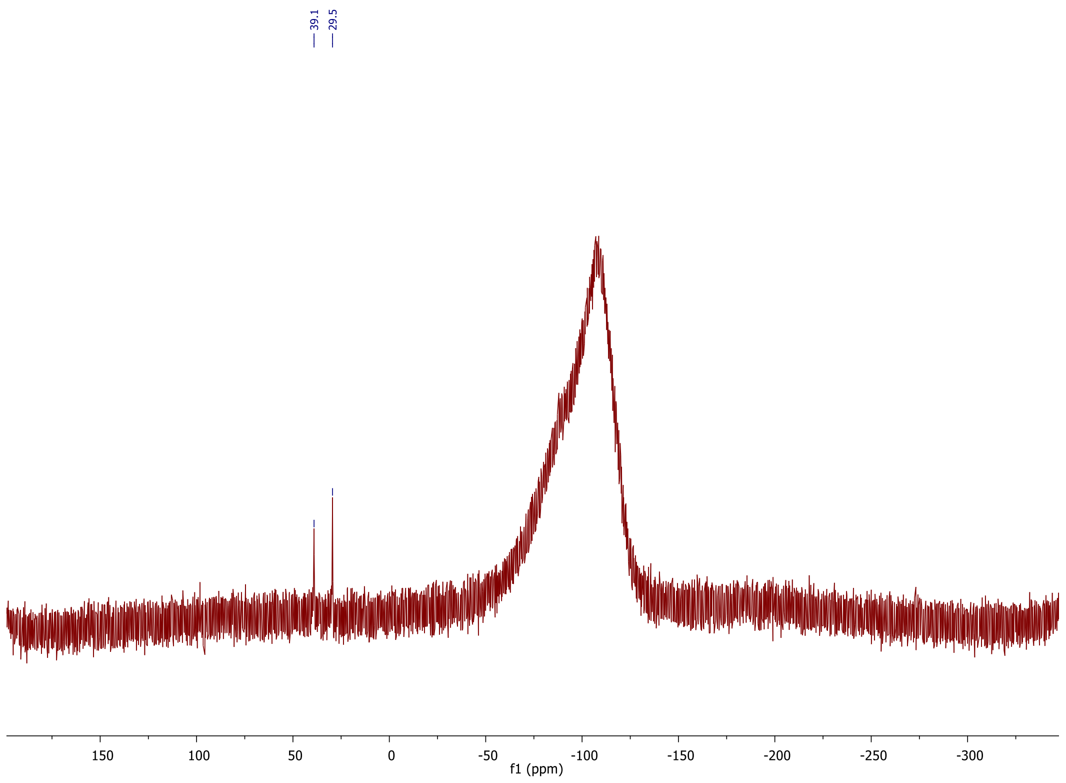

Figure S27 $^{29}Si\{^1H\}$ NMR spectrum of **5b** in C_6D_6 under 1 bar N_2 at 298 K.

A4 High Resolution Mass Spectrometry

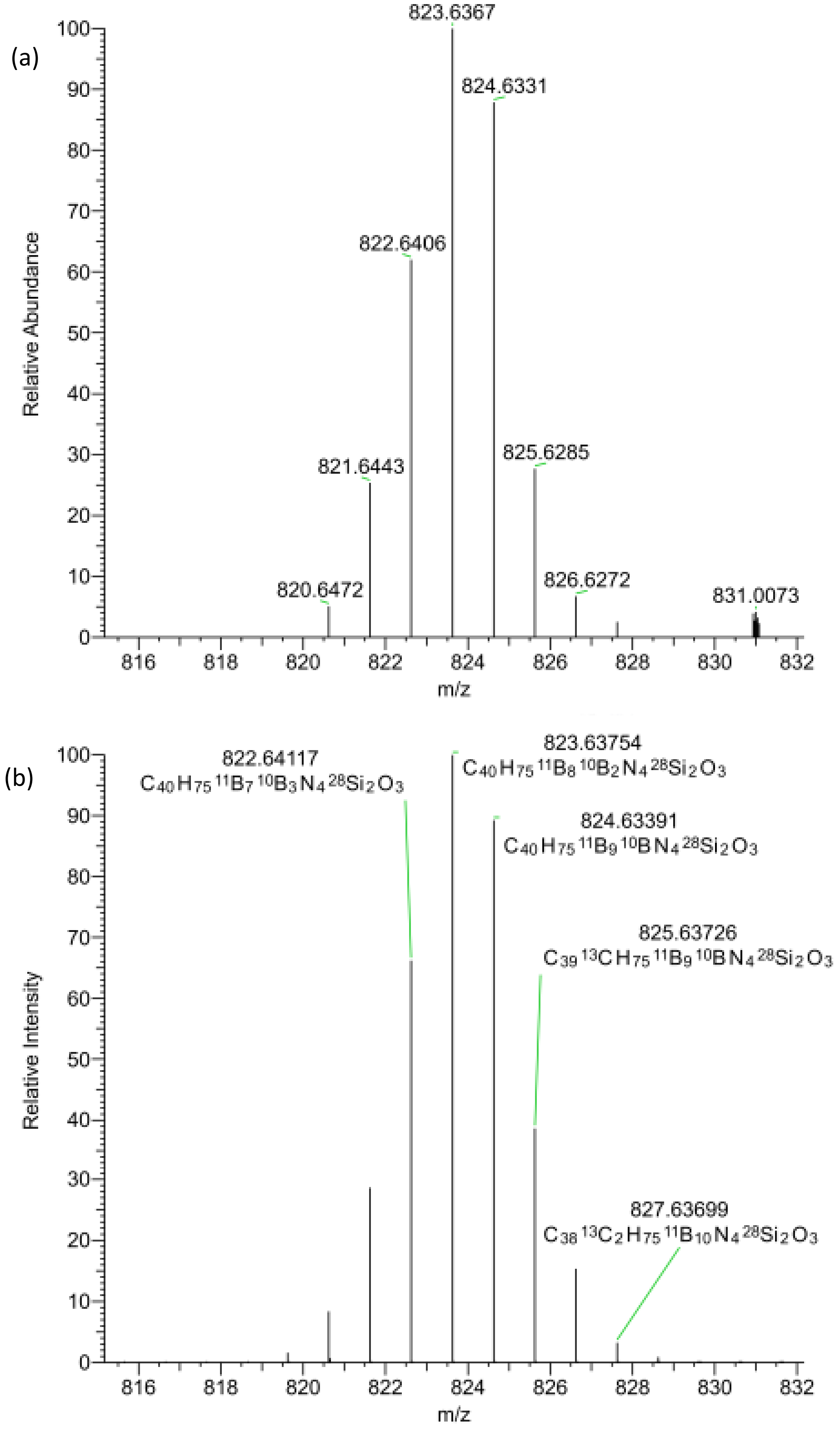

Figure S28 (a) Experimental MS Spectrum of **1b** (b) Predicted MS Spectrum of **1b**-derived fragment

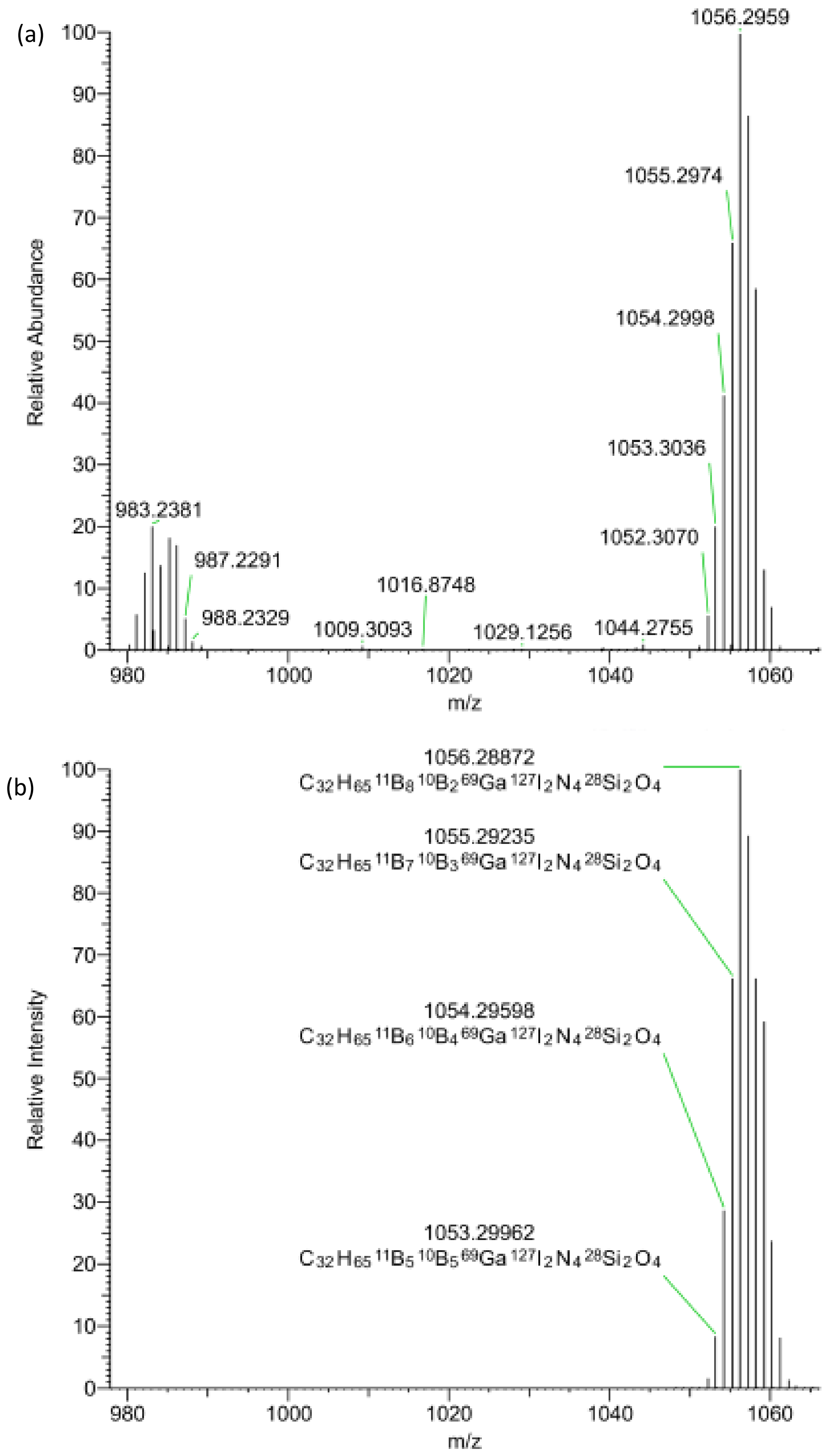

Figure S29 (a) Experimental MS Spectrum of **4b** (b) Predicted MS Spectrum of **4b**-derived fragment

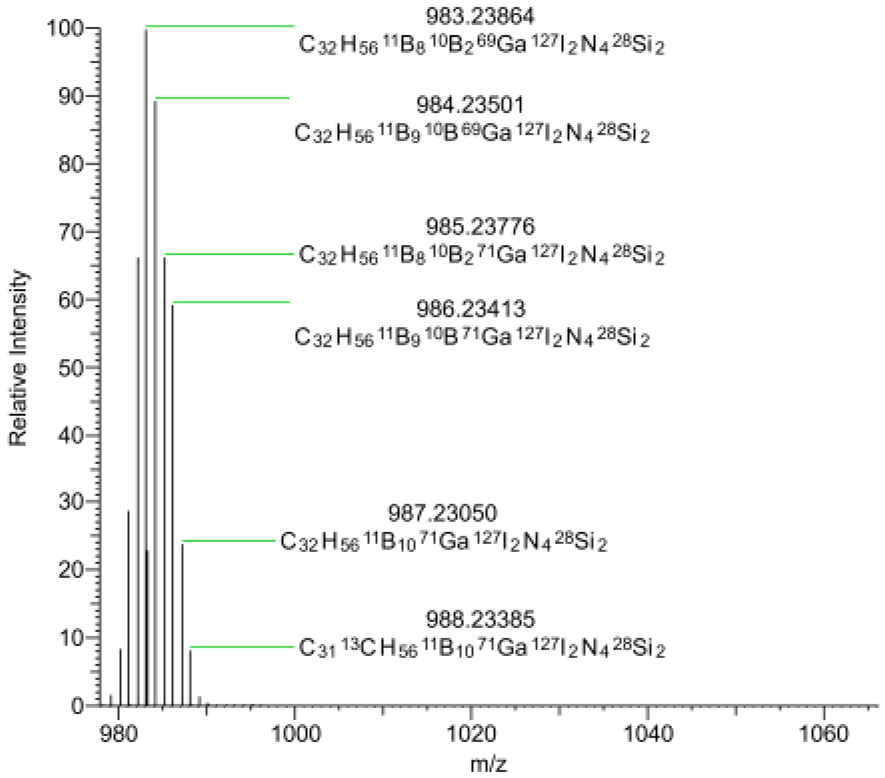

Figure S30 Predicted MS Spectrum of **4b**

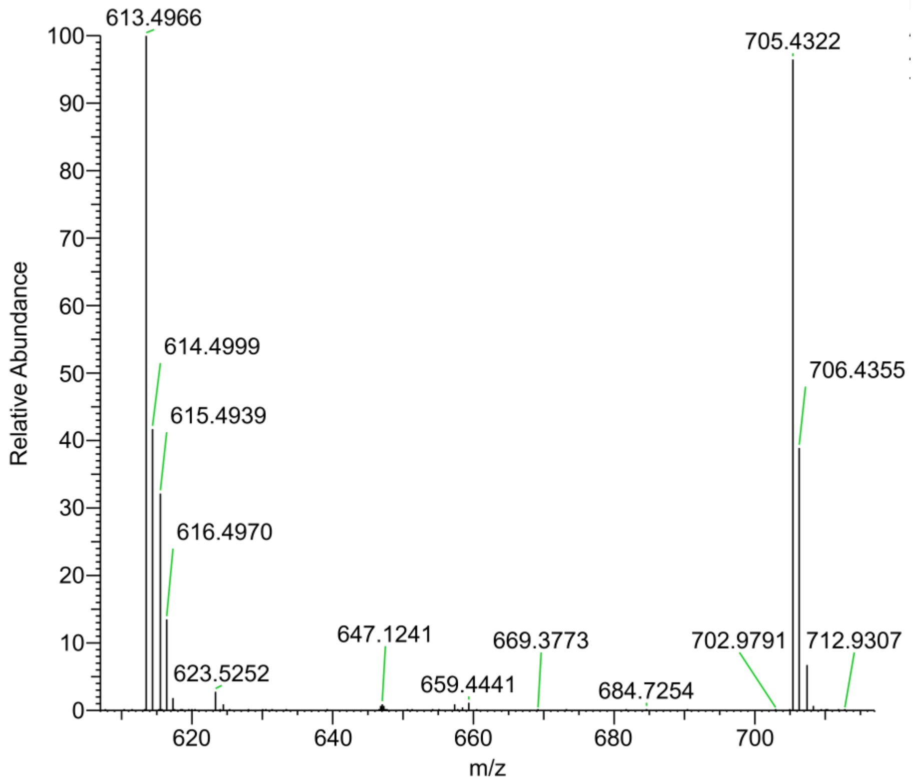

Figure S31 Experimental MS Spectrum of **5b**

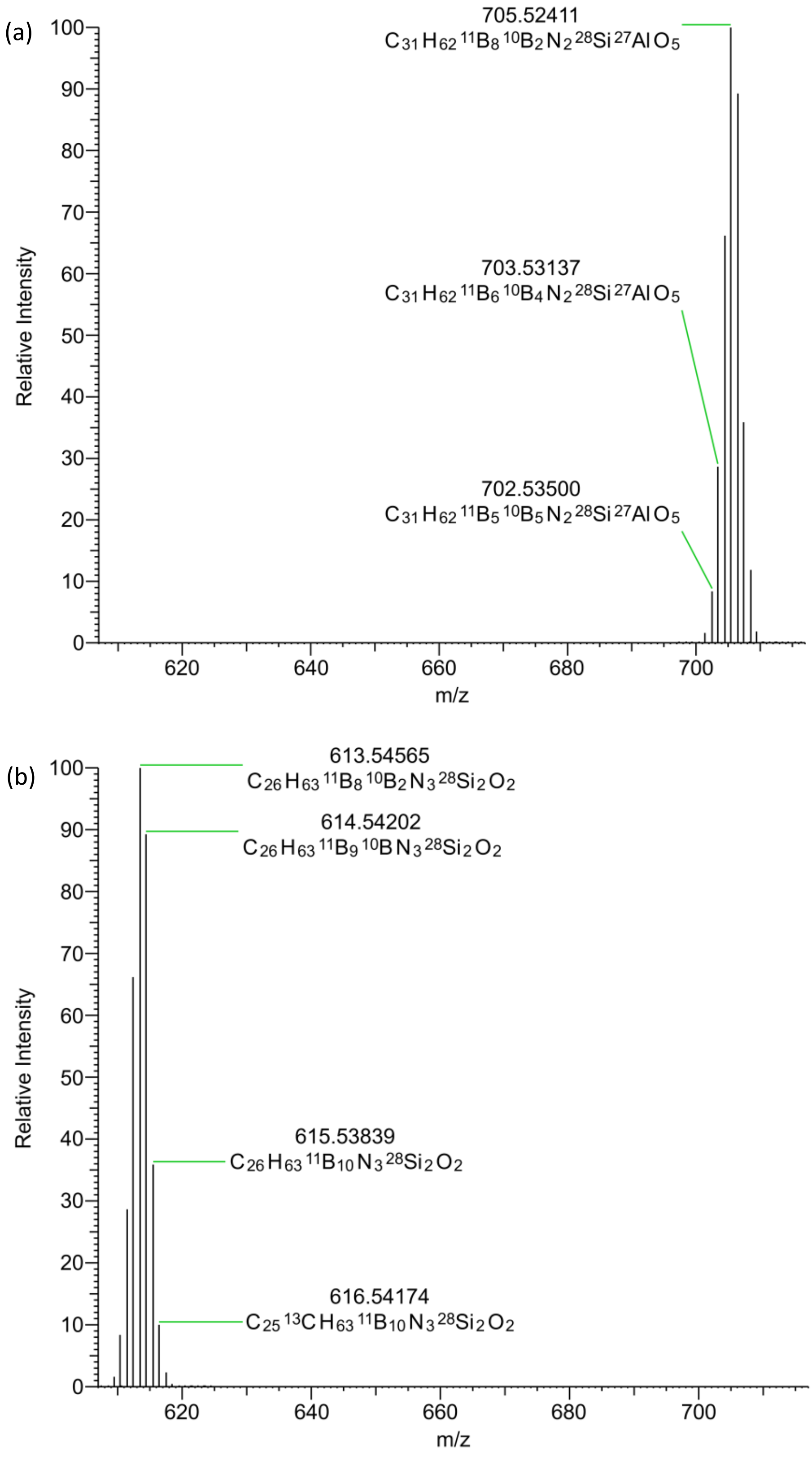

Figure S32 (a) and (b) Predicted MS Spectra of **5b**-derived fragments

A5 Single Crystal X-Ray Diffraction Data

Table S1 Crystallographic Data and Structure Refinement for **2b**

Empirical formula	C32 H56 Al2 B10 I6 N4 Si2	
CCDC number	2418300	
Crystal description	Colorless prism	
Formula weight	1476.44	
Temperature (K)	150.0	
Wavelength (Å)	1.54184	
Crystal system	Monoclinic	
Space group	$P2_1/n$	
Unit cell dimensions (Å or °)	a = 14.4094(4)<br>b = 17.2943(4)<br>c = 23.7145(6)	α = 90<br>β = 92.257(3)<br>γ = 90
Volume (Å^3)	5905.1(3)	
Z	4	
Density ρ (calculated, g cm^{-3})	1.661	
Absorption coefficient μ (mm^{-1})	25.646	
F(000)	2792	
Crystal size (mm^3)	0.21 x 0.11 x 0.07	
Theta range for data collection (°)	3.164 to 73.095	
Index ranges	-16 ≤ h ≤ 17<br>-20 ≤ k ≤ 21<br>-28 ≤ l ≤ 29	
Reflections collected	42439	
Independent reflections	11605 [R(int) = 0.0656]	
Completeness to theta = 67.684°	1.000	
Absorption correction	Semi-empirical from equivalents	
Max. and min. transmission	1.00000<br>0.11885	
Refinement method	Full-matrix least-squares on F^2	
Data / restraints / parameters	11605 / 24 / 576	
Goodness-of-fit on F^2	1.020	
Final R indices [I>2sigma(I)]	R1 = 0.0489, wR2 = 0.1138	
R indices (all data)	R1 = 0.0787, wR2 = 0.1288	
Extinction coefficient	n/a	
Largest diff. peak and hole (e.Å^{-3})	1.043<br>-0.966	

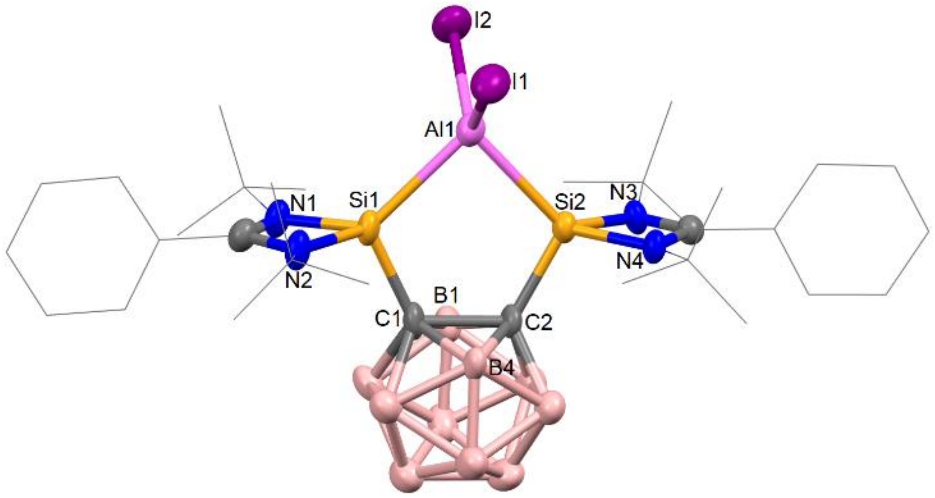

Figure S33 Molecular Structure of **2b**-cation. Thermal ellipsoids are drawn at the 50% level. $[AlI_4]$ counterion, H atoms and solvent molecules omitted for clarity.

Table S2 Selected interatomic distances and angles of **2b**.

Interatomic distances (Å)		Angles (°)	
Al(1)-I(1)	2.497(2)	I(1)-Al(1)-I(2)	113.37(7)
Al(1)-I(2)	2.510(2)	Si(1)-Al(1)-Si(2)	89.07(8)
Al(1)-Si(1)	2.496(3)	Si(1)-Al(1)-I(1)	112.26(9)
Al(1)-Si(2)	2.490(2)	Si(2)-Al(1)-I(1)	113.63(8)
Si(1)-N(1)	1.808(5)	C(1)-Si(1)-Al(1)	108.12(19)
Si(1)-N(2)	1.808(5)	C(2)-Si(2)-Al(1)	107.94(19)
Si(2)-N(3)	1.795(5)	Al(1)-Si(1)-N(1)	127.23(19)
Si(2)-N(4)	1.801(5)	Al(1)-Si(1)-N(2)	127.24(18)
Si(1)-C(1)	1.914(6)	Al(1)-Si(2)-N(3)	127.09(18)
Si(2)-C(2)	1.923(6)	Al(1)-Si(2)-N(4)	125.89(17)
C(1)-C(2)	1.729(8)	N(1)-Si(1)-N(2)	73.5(2)
C(1)-B(1)	1.700(9)	N(3)-Si(2)-N(4)	73.4(2)
C(1)-B(4)	1.713(9)	Si(1)-C(1)-C(2)	117.4(4)
C(2)-B(1)	1.710(10)	Si(2)-C(2)-C(1)	117.5(4)
C(2)-B(4)	1.705(9)		

Table S3 Crystallographic Data and Structure Refinement for [**3**(OEt_2)$_4$]

Empirical formula	C48 H96 Al B10 I2 K N4 O4 Si2	
CCDC number	2418301	
Crystal description	Red block	
Formula weight	1277.44	
Temperature (K)	150(2)	
Wavelength (Å)	1.54184	
Crystal system	Monoclinic	
Space group	$P2_1/n$	
Unit cell dimensions (Å or °)	a = 14.7194(3)<br>b = 30.6034(7)<br>c = 15.2868(3)	α = 90<br>β = 94.863(2)<br>γ = 90
Volume (Å^3)	6861.4(3)	
Z	4	
Density ρ (calculated, g cm^{-3})	1.237	
Absorption coefficient μ (mm^{-1})	8.492	
F(000)	2640	
Crystal size (mm^3)	0.23 x 0.17 x 0.09	
Theta range for data collection (°)	2.888 to 67.495	
Index ranges	-17 ≤ h ≤ 17<br>-25 ≤ k ≤ 36<br>-18 ≤ l ≤ 16	
Reflections collected	26143	
Independent reflections	12351 [R(int) = 0.0381]	
Completeness to theta = 67.684°	0.999	
Absorption correction	Semi-empirical from equivalents	
Max. and min. transmission	1.00000<br>0.69472	
Refinement method	Full-matrix least-squares on F^2	
Data / restraints / parameters	12351 / 148 / 669	
Goodness-of-fit on F^2	1.010	
Final R indices [I>2sigma(I)]	R1 = 0.0491, wR2 = 0.1228	
R indices (all data)	R1 = 0.0664, wR2 = 0.1372	
Extinction coefficient	n/a	
Largest diff. peak and hole (e.Å^{-3})	1.238<br>-0.904	

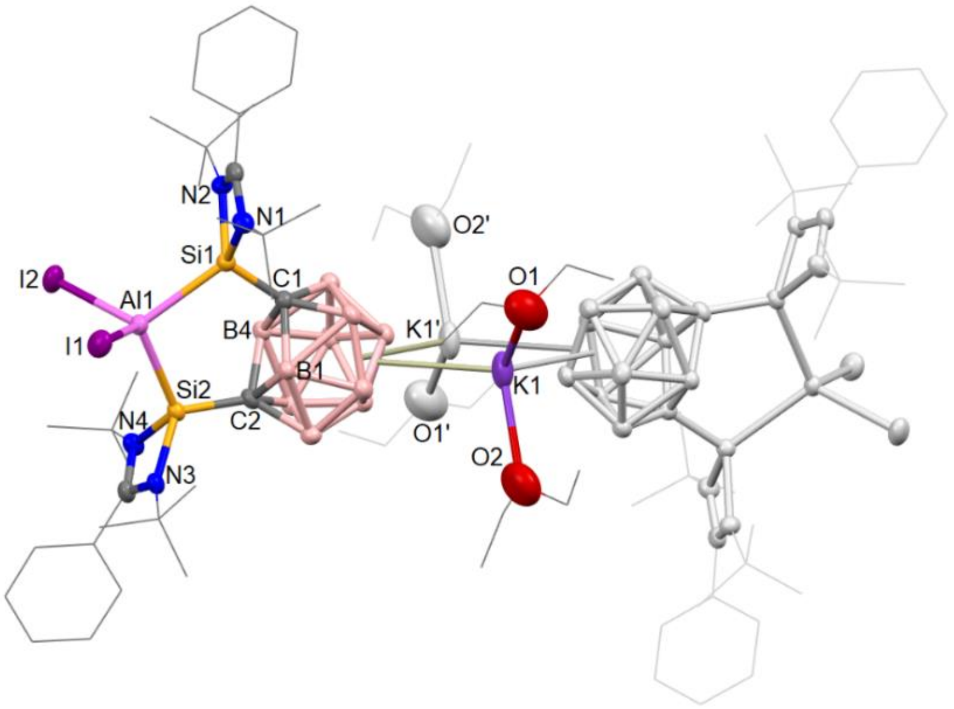

Figure S34 Molecular Structure of [**3**(OEt$_2$)$_4$]. Thermal ellipsoids are drawn at the 50% level. H atoms and solvent molecules omitted for clarity.

Table S4 Selected interatomic distances and angles of [**3**(OEt$_2$)$_4$].

Interatomic distances (Å)		Angles (°)	
Al(1)-I(1)	2.5688(13)	I(1)-Al(1)-I(2)	103.62(4)
Al(1)-I(2)	2.5786(13)	Si(1)-Al(1)-Si(2)	101.99(6)
Al(1)-Si(1)	2.4950(17)	Si(1)-Al(1)-I(1)	110.60(6)
Al(1)-Si(2)	2.5060(17)	Si(2)-Al(1)-I(1)	115.49(6)
Si(1)-N(1)	1.830(4)	C(1)-Si(1)-Al(1)	107.89(15)
Si(1)-N(2)	1.836(4)	C(2)-Si(2)-Al(1)	107.63(15)
Si(2)-N(3)	1.833(4)	Al(1)-Si(1)-N(1)	120.35(13)
Si(2)-N(4)	1.834(4)	Al(1)-Si(1)-N(2)	121.45(13)
Si(1)-C(1)	1.794(4)	Al(1)-Si(2)-N(3)	120.36(13)
Si(2)-C(2)	1.795(4)	Al(1)-Si(2)-N(4)	120.25(14)
C(1)-B(1)	1.684(6)	N(1)-Si(1)-N(2)	71.96(17)
C(1)-B(4)	1.698(7)	N(3)-Si(2)-N(4)	71.59(17)
C(2)-B(1)	1.698(7)	C(1)-B(1)-C(2)	99.8(3)
C(2)-B(4)	1.691(6)	C(1)-B(4)-C(2)	99.5(3)
C(1)···C(2)	2.587	Si(1)-C(1)···C(2)	111.15
K(1)···cage	3.188	Si(2)-C(2)···C(1)	111.31
K(1')···cage	3.166	cage···K(1)···cage	111.17

Table S5 Crystallographic Data and Structure Refinement for **4a**

Empirical formula	C46 H72 Al B10 I2 N4 Si2	
CCDC number	2418298	
Crystal description	Red prism	
Formula weight	1126.13	
Temperature (K)	150.15	
Wavelength (Å)	1.54184	
Crystal system	Triclinic	
Space group	P-1	
Unit cell dimensions (Å or °)	a = 14.6482(7)<br>b = 14.8290(7)<br>c = 18.4781(10)	α = 105.669(4)<br>β = 106.625(5)<br>γ = 100.731(4)
Volume (Å^3)	3548.9(3)	
Z	2	
Density ρ (calculated, g cm^{-3})	1.054	
Absorption coefficient μ (mm^{-1})	7.600	
F(000)	1146	
Crystal size (mm^3)	0.19 x 0.14 x 0.11	
Theta range for data collection (°)	3.230 to 72.972	
Index ranges	-17 ≤ h ≤ 18<br>-18 ≤ k ≤ 15<br>-21 ≤ l ≤ 22	
Reflections collected	26771	
Independent reflections	13734 [R(int) = 0.0611]	
Completeness to theta = 67.684°	0.998	
Absorption correction	Semi-empirical from equivalents	
Max. and min. transmission	1.00000<br>0.30943	
Refinement method	Full-matrix least-squares on F^2	
Data / restraints / parameters	13734 / 0 / 588	
Goodness-of-fit on F^2	0.975	
Final R indices [I>2sigma(I)]	R1 = 0.0534, wR2 = 0.1353	
R indices (all data)	R1 = 0.0753, wR2 = 0.1537	
Extinction coefficient	n/a	
Largest diff. peak and hole (e.Å^{-3})	1.290<br>-1.384	

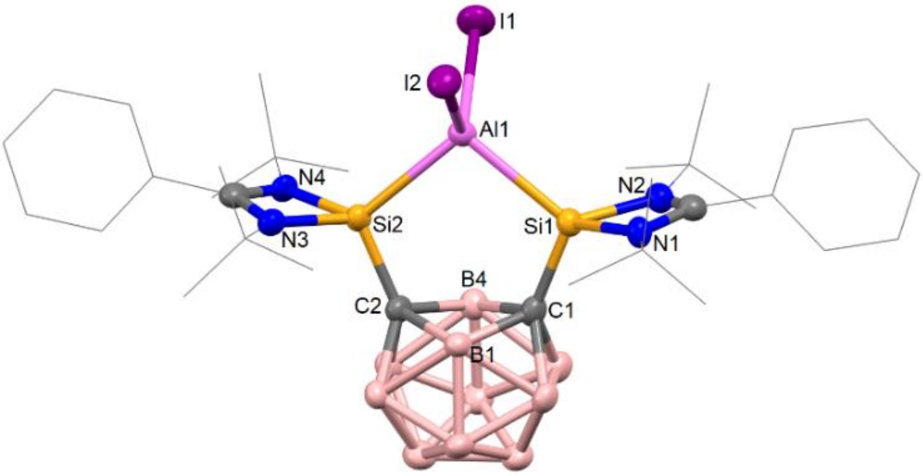

Figure S35 Molecular Structure of **4a**. Thermal ellipsoids are drawn at the 50% level. H atoms and solvent molecules omitted for clarity.

Table S6 Selected interatomic distances and angles of **4a**

Interatomic distances (Å)		Angles (°)	
Al(1)-I(1)	2.5466(13)	I(1)-Al(1)-I(2)	106.37(5)
Al(1)-I(2)	2.5448(14)	Si(1)-Al(1)-Si(2)	99.84(6)
Al(1)-Si(1)	2.5046(17)	Si(1)-Al(1)-I(1)	113.15(6)
Al(1)-Si(2)	2.4901(17)	Si(2)-Al(1)-I(1)	112.99(5)
Si(1)-N(1)	1.823(3)	C(1)-Si(1)-Al(1)	107.45(14)
Si(1)-N(2)	1.818(4)	C(2)-Si(2)-Al(1)	107.84(14)
Si(2)-N(3)	1.815(4)	Al(1)-Si(1)-N(1)	123.03(13)
Si(2)-N(4)	1.818(3)	Al(1)-Si(1)-N(2)	123.58(13)
Si(1)-C(1)	1.823(5)	Al(1)-Si(2)-N(3)	122.08(13)
Si(2)-C(2)	1.831(4)	Al(1)-Si(2)-N(4)	122.48(13)
C(1)-B(1)	1.733(7)	N(1)-Si(1)-N(2)	72.31(16)
C(1)-B(4)	1.742(6)	N(3)-Si(2)-N(4)	72.56(17)
C(2)-B(1)	1.739(6)	C(1)-B(1)-C(2)	88.8(3)
C(2)-B(4)	1.714(7)	C(1)-B(4)-C(2)	89.4(3)
C(1)···C(2)	2.431--	Si(1)-C(1)···C(2)	112.58
		Si(2)-C(2)···C(1)	112.16

Table S7 Crystallographic Data and Structure Refinement for **4b**

Empirical formula	C32 H56 Ga B10 I2 N4 Si2	
CCDC number	2418297	
Crystal description	Orange block	
Formula weight	984.60	
Temperature (K)	150(2)	
Wavelength (Å)	0.71073	
Crystal system	Orthorhombic	
Space group	Cmcm	
Unit cell dimensions (Å or °)	a = 23.2436(3)<br>b = 18.9817(3)<br>c = 13.9956(2)	α = 90<br>β = 90<br>γ = 90
Volume (Å^3)	6174.90(15)	
Z	4	
Density ρ (calculated, g cm^{-3})	1.059	
Absorption coefficient μ (mm^{-1})	1.505	
F(000)	1964	
Crystal size (mm^3)	0.21 x 0.14 x 0.09	
Theta range for data collection (°)	1.752 to 26.109	
Index ranges	-26 ≤ h ≤ 28<br>-22 ≤ k ≤ 23<br>-10 ≤ l ≤ 17	
Reflections collected	12678	
Independent reflections	3236 [R(int) = 0.0321]	
Completeness to theta = 67.684°	0.996	
Absorption correction	Semi-empirical from equivalents	
Max. and min. transmission	1.00000<br>0.29306	
Refinement method	Full-matrix least-squares on F^2	
Data / restraints / parameters	3236 / 13 / 140	
Goodness-of-fit on F^2	1.065	
Final R indices [I>2sigma(I)]	R1 = 0.0380, wR2 = 0.1049	
R indices (all data)	R1 = 0.0418, wR2 = 0.1084	
Extinction coefficient	n/a	
Largest diff. peak and hole (e.Å^{-3})	0.469<br>-1.359	

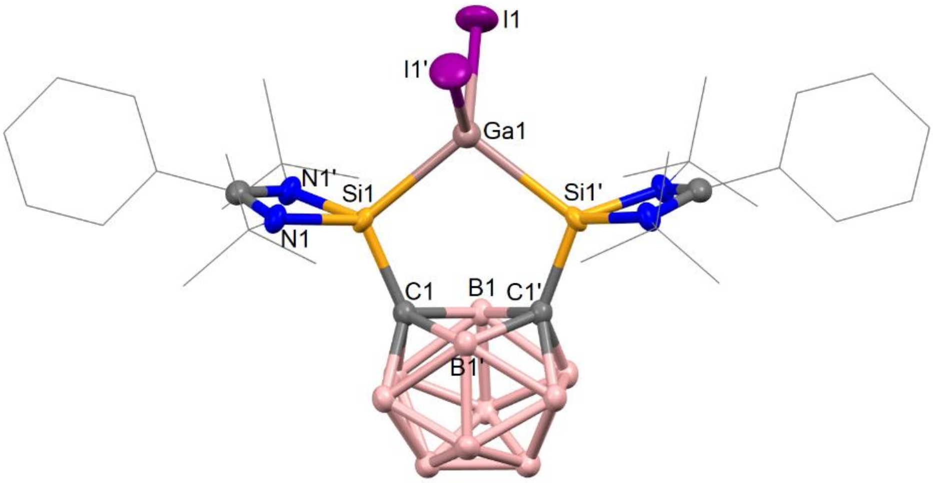

Figure S36 Molecular Structure of **4b**. Thermal ellipsoids are drawn at the 50% level. H atoms and solvent molecules omitted for clarity.

Table S8 Selected interatomic distances and angles of **4b**.

Interatomic distances (Å)		Angles (°)	
Ga(1)-I(1)	2.5638(5)	I(1)-Ga(1)-I(1')	102.21(3)
Ga(1)-Si(1)	2.4331(11)	Si(1)-Ga(1)-Si(1')	101.55(5)
Si(1)-N(1)	1.806(2)	Si(1)-Ga(1)-I(1)	113.395(12)
Si(1)-C(1)	1.821(4)	C(1)-Si(1)-Ga(1)	107.38(12)
C(1)-B(1)	1.726(4)	Ga(1)-Si(1)-N(1)	120.82(8)
C(1)···C(1')	2.415	N(1)-Si(1)-N(1')	72.94(15)
		C(1)-B(1)-C(1')	88.8(2)
		Si(1)-C(1)···C(1')	111.84

Table S9 Crystallographic Data and Structure Refinement for [**5b**(OEt_2)$_4$]

Empirical formula	C58 H100 Al B10 K N4 O2 Si2	
CCDC number	2418302	
Crystal description	Orange prism	
Formula weight	1115.17	
Temperature (K)	150.15	
Wavelength (Å)	1.54184	
Crystal system	Monoclinic	
Space group	$P2_1/c$	
Unit cell dimensions (Å or °)	a = 21.7751(10)<br>b = 15.1015(3)<br>c = 24.8744(9)	α = 90<br>β = 109.723(4)<br>γ = 90
Volume (Å^3)	7699.8(5)	
Z	4	
Density ρ (calculated, g cm^{-3})	0.963	
Absorption coefficient μ (mm^{-1})	1.276	
F(000)	2408	
Crystal size (mm^3)	0.16 x 0.10 x 0.08	
Theta range for data collection (°)	3.483 to 67.500	
Index ranges	$-26 \leq h \leq 26$<br>$-17 \leq k \leq 18$<br>$-23 \leq l \leq 29$	
Reflections collected	54462	
Independent reflections	13867 [R(int) = 0.0847]	
Completeness to theta = 67.684°	0.999	
Absorption correction	Semi-empirical from equivalents	
Max. and min. transmission	1.00000<br>0.38121	
Refinement method	Full-matrix least-squares on F^2	
Data / restraints / parameters	13867 / 132 / 812	
Goodness-of-fit on F^2	1.021	
Final R indices [I>2sigma(I)]	R1 = 0.0845, wR2 = 0.2126	
R indices (all data)	R1 = 0.1177, wR2 = 0.2445	
Extinction coefficient	n/a	
Largest diff. peak and hole (e.Å^{-3})	0.894<br>-0.457	

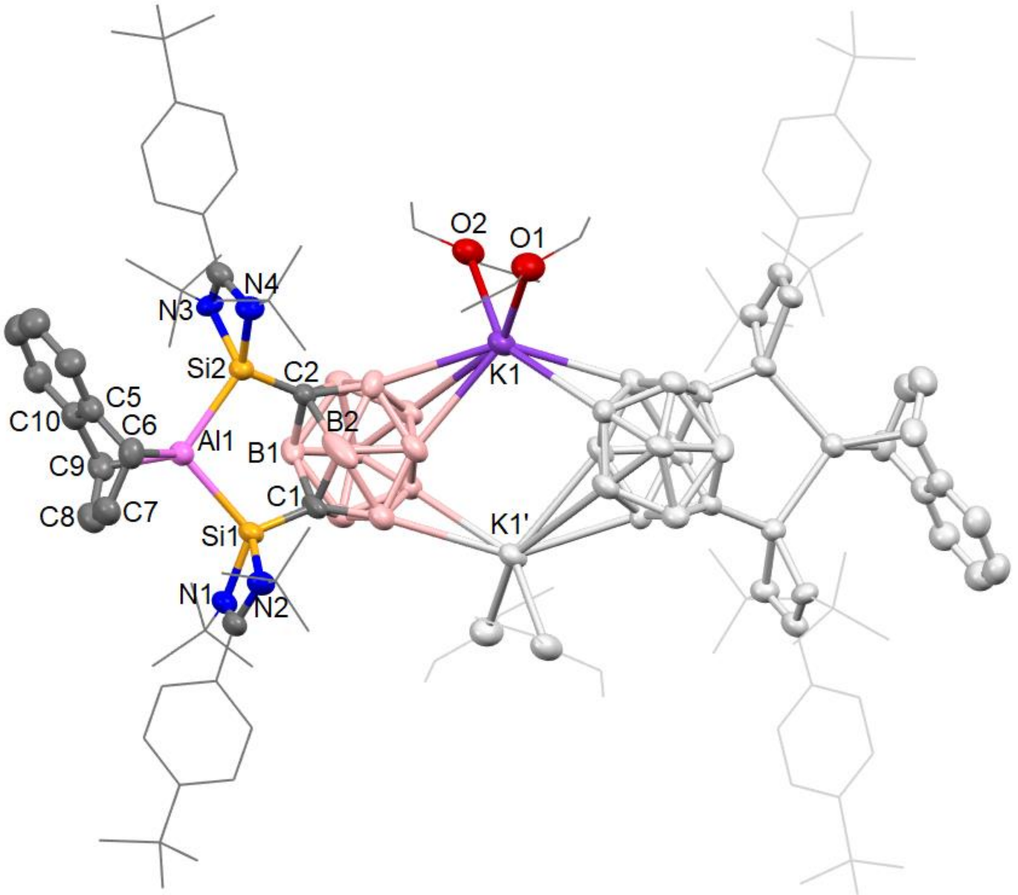

Figure S37 Molecular Structure of [**5b**(OEt_2)$_4$]. Thermal ellipsoids are drawn at the 50% level. H atoms and solvent molecules omitted for clarity.

Table S10 Selected interatomic distances and angles of [**5b**(OEt_2)$_4$].

Interatomic distances (Å)		Angles (°)	
Al(1)-C(6)	2.056(4)	C(6)-Al(1)-C(9)	77.49(17)
Al(1)-C(9)	2.066(4)	Si(1)-Al(1)-Si(2)	100.95(5)
Al(1)-Si(1)	2.5010(15)	Si(1)-Al(1)-C(6)	119.30(12)
Al(1)-Si(2)	2.5253(15)	Si(2)-Al(1)-C(6)	121.48(12)
C(6)-C(5)	1.480(6)	C(5)-C(6)-C(7)	109.6(3)
C(6)-C(7)	1.527(6)	C(8)-C(9)-C(10)	109.5(4)
C(7)-C(8)	1.339(7)	C(6)-C(7)-C(8)	113.2(4)
C(5)-C(10)	1.414(6)	C(7)-C(8)-C(9)	115.2(4)
C(9)-C(10)	1.493(6)	C(1)-Si(1)-Al(1)	108.4(3)
C(9)-C(8)	1.503(6)	C(2)-Si(2)-Al(1)	107.87(12)
Si(1)-N(1)	1.838(3)	Al(1)-Si(1)-N(1)	118.86(12)
Si(1)-N(2)	1.834(4)	Al(1)-Si(1)-N(2)	117.41(13)
Si(2)-N(3)	1.843(3)	Al(1)-Si(2)-N(3)	121.10(12)
Si(2)-N(4)	1.848(3)	Al(1)-Si(2)-N(4)	120.60(12)

Si(1)-C(1)	1.847(9)	N(1)-Si(1)-N(2)	71.30(15)
Si(2)-C(2)	1.799(4)	N(3)-Si(2)-N(4)	70.92(14)
C(1)-B(1)	1.774(14)	C(1)-B(1)-C(2)	99.1(5)
C(1)-B(2)	1.678(10)	C(1)-B(2)-C(2)	99.6(5)
C(2)-B(1)	1.643(6)	Si(1)-C(1)···C(2)	109.30
C(2)-B(2)	1.727(12)	Si(2)-C(2)···C(1)	111.66
C(1)···C(2)	2.602		

References

[1] G. M. Sheldrick, *Acta Crystallogr. Sect. C Struct. Chem.* **2015**, *71*, 3–8.
[2] O. V. Dolomanov, L. J. Bourhis, R. J. Gildea, J. A. K. Howard, H. Puschmann, *J. Appl. Crystallogr.* **2009**, *42*, 339–341.
[3] L. J. Bourhis, O. V. Dolomanov, R. J. Gildea, J. A. K. Howard, H. Puschmann, *Acta Crystallogr. Sect. Found. Adv.* **2015**, *71*, 59–75.
[4] S. Stoll, A. Schweiger, *J. Magn. Reson.* **2006**, *178*, 42–55.
[5] D. J. Hirsh, G. W. Brudvig, *Nat. Protoc.* **2007**, *2*, 1770–1781.
[6] Y.-P. Zhou, S. Raoufmoghaddam, T. Szilvási, M. Driess, *Angew. Chem. Int. Ed.* **2016**, *55*, 12868–12872.
[7] S. S. Sen, H. W. Roesky, D. Stern, J. Henn, D. Stalke, *J. Am. Chem. Soc.* **2010**, *132*, 1123–1126.
[8] C. Shan, S. Dong, S. Yao, J. Zhu, M. Driess, *J. Am. Chem. Soc.* **2023**, *145*, 7084–7089.
[9] T. A. Scott, B. A. Ooro, D. J. Collins, M. Shatruk, A. Yakovenko, K. R. Dunbar, H.-C. Zhou, *Chem Commun* **2009**, 65–67.
[10] J. Hicks, M. Juckel, A. Paparo, D. Dange, C. Jones, *Organometallics* **2018**, *37*, 4810–4813.
[11] V. Lavallo, Y. Canac, C. Präsang, B. Donnadieu, G. Bertrand, *Angew. Chem. Int. Ed.* **2005**, *44*, 5705–5709.
[12] F. J. M. Gil, M. A. Salgado, J. M. Gil, *Synth. React. Inorg. Met.-Org. Chem.* **1986**, *16*, 663–666.
[13] J. C. Beamish, M. Wilkinson, I. J. Worrall, *Inorg. Chem.* **1978**, *17*, 2026–2027.
[14] J. C. Beamish, A. Boardman, I. J. Worrall, *Polyhedron* **1991**, *10*, 95–99.
[15] S. K. Mellerup, Y. Cui, F. Fantuzzi, P. Schmid, J. T. Goettel, G. Bélanger-Chabot, M. Arrowsmith, I. Krummenacher, Q. Ye, V. Engel, B. Engels, H. Braunschweig, *J. Am. Chem. Soc.* **2019**, *141*, 16954–16960.
[16] D. Dhara, L. Endres, I. Krummenacher, M. Arrowsmith, R. D. Dewhurst, B. Engels, R. Bertermann, M. Finze, S. Demeshko, F. Meyer, F. Fantuzzi, H. Braunschweig, *Angew. Chem. Int. Ed.* **2024**, *63*, e202401052.
[17] X. Chen, D. Yang, F. Cao, Z. Mo, *J. Am. Chem. Soc.* **2024**, *146*, 29278–29284.
